# Identification and validation of a five-lncRNA signature for predicting survival with targeted drug candidates in ovarian cancer

**DOI:** 10.1080/21655979.2021.1946632

**Published:** 2021-07-05

**Authors:** Nuan Lin, Jia-zhe Lin, Yoshiaki Tanaka, Pingnan Sun, Xiaoling Zhou

**Affiliations:** a Obstetrics & Gynecology Department, The First Affiliated Hospital of Shantou University Medical College, Shantou, People’s Republic of China; b Stem Cell Research Center, Shantou University Medical College, Shantou, People’s Republic of China; c The Center for Reproductive Medicine, Shantou University Medical College, Shantou, People’s Republic of China; d Guangdong Provincial Key Laboratory of Infectious Diseases and Molecular Immunopathology, Shantou University Medical College, Shantou, People’s Republic of China; e Neurosurgical Department, The First Affiliated Hospital of Shantou University Medical College, Shantou, People’s Republic of China; f Department of Genetics, Yale Stem Cell Center, Yale School of Medicine, New Haven, CT, USA

**Keywords:** Ovarian cancer, risk signature, long non-coding rnas, small molecular drugs, computational biology

## Abstract

The dysregulation of long non-coding RNAs (lncRNAs) plays a crucial role in ovarian cancer (OC). In this study, we screened out five differentially expressed lncRNAs *(AC092718.4, AC138035.1, BMPR1B-DT, RNF157-AS1,* and *TPT1-AS1)* between OC and normal ovarian based on TCGA and GTEx RNA-seq databases by using Kaplan–Meier analysis and univariate Cox, LASSO, and multivariate Cox regression. Then, a risk signature was constructed, with 1, 3, 5-year survival prediction accuracy confirmed by ROC curves, and an online survival calculator for easier clinical use. With lncRNA-microRNA-mRNA regulatory networks established, Gene Ontology (GO) and Kyoto Encyclopedia of Genes and Genomes (KEGG) analyses were performed, suggesting the involvement of a variety of cancer-related functions and pathways. Finally, five candidate small-molecule drugs (thioridazine, trifluoperazine, loperamide, LY294002, and puromycin) were predicted by Connectivity Map. In conclusion, we identified a 5-lncRNA signature of prognostic value with its ceRNA networks, and five candidate drugs against OC.

**Figure:**
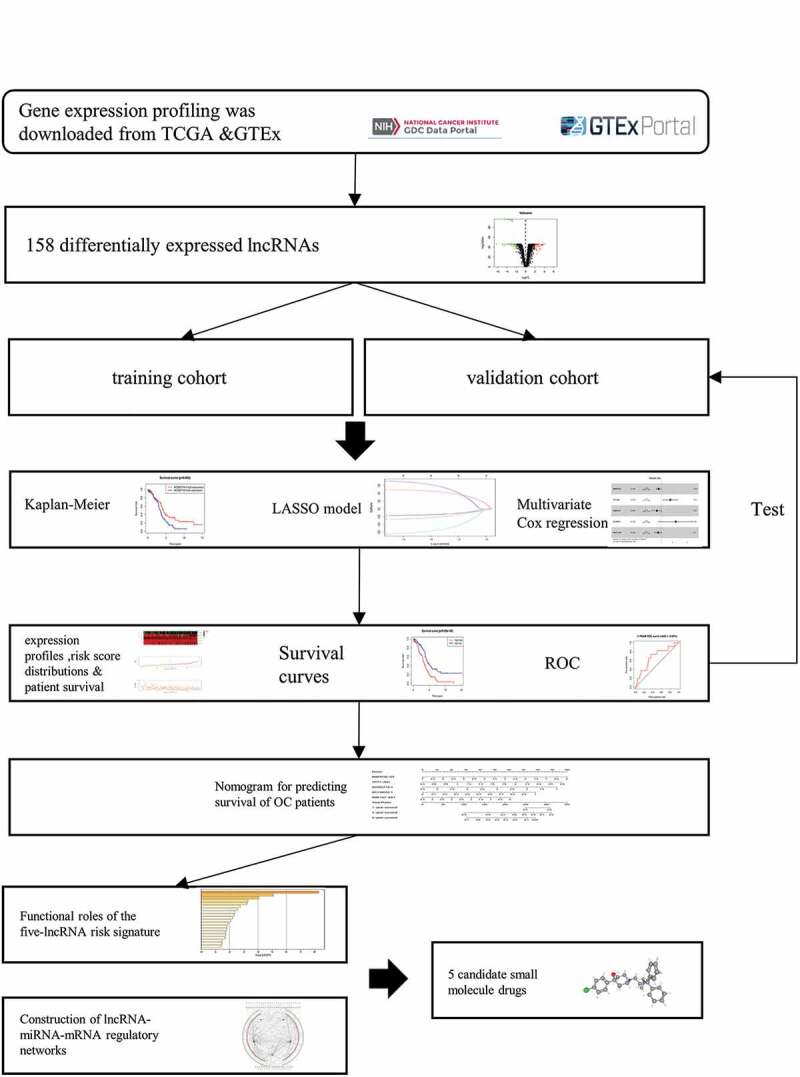

## 1. Introduction

Ovarian cancer (OC) represents the seventh most common cancer among women in the world and is the most lethal gynecological malignancy with a 5-year survival of 46% **[**1**]**. Current first-line treatment for OC patients involves primary debulking surgery (PDS) for all International Federation of Gynecology and Obstetrics (FIGO) stages, followed by combination chemotherapy, usually carboplatin and paclitaxel in advanced-stage OC **[**1,2**]**. Unfortunately, the efficacy of these treatments is limited, and the prognosis of OC patients is still poor, with a 5-year survival of only 20–40% for stage III or IV cancer patients who received surgery and chemotherapy **[**3**]**. A majority of patients either undergo relapse or succumb to the disease due to chemotherapy resistance **[**3,4**]**, which is attributed to a wide range of metabolic or structural properties within tumor cells, such as multidrug resistance proteins, mismatch repair processes, and alterations in the p53 pathway **[**5**]**. In addition, about 75% of patients are diagnosed at an advanced stage because of the asymptomatic nature of OC **[**1**]**. Thus, we explored novel molecular biomarkers for diagnosis, prognosis, and targeted therapy for OC.

Advances in sequencing technologies have led to the discovery of non-coding RNAs, which are key regulators of physiological activities and pathological processes, particularly in cancer **[**6**]**. Long non-coding RNAs (lncRNAs) are widely expressed and can ultimately affect gene expression in diverse biological and physiopathological contexts by functioning as competing endogenous RNAs (ceRNAs) **[**7**]**. CeRNAs, also known as miRNA ‘decoy’ or miRNA‘sponges’, are RNA transcripts that compete for the binding to miRNA via base pairing with miRNA recognition/response elements (MREs), resulting in subsequent reduction of the amount of available miRNAs to target messenger RNAs (mRNAs) **[**8,9**]**. Since human diseases are driven by complex interactions among a variety of molecular mediators rather than being caused by single molecular defect, ceRNA networks hold the promise of uncovering the causes and revolutionizing the diagnosis and treatments of human diseases **[**10**]**. For instance, lncRNA PVT1 plays an important role in human breast cancer by dysregulating the ceRNA-ceRNA network **[**11**]**. LncRNA-mediated ceRNA networks are involved in colorectal cancer initiation, progression, and metastasis **[**12**]**. Zhao et al. showed that a lncRNA SNHG5/miR-32 axis plays an important role in the proliferation and migration of gastric cancer by targeting KLF4 **[**13**]**.

Therefore, we hypothesized that some lncRNAs can serve as critical biomarkers as well as therapeutic targets for OC. Hence, in this study, we aimed to develop a prognostic lncRNA signature with predicted candidate drugs for OC. We first identified and validated five differentially expressed lncRNAs (DElncRNA) for the development of a risk signature for OC patient survival. CeRNA regulatory networks were further established, as well as the relevant biological functions and pathways identified and candidate drugs predicted based on the prognostic signature. Finally, an online survival calculator was established to facilitate clinical practitioners.

## 2. Materials and methods

### 2.1. Data set extraction and processing

Functional genomic data sets were downloaded from UCSC Xena, the RNA sequencing profiles, clinical information, and survival data from serous OC (dataset ID: TCGA-OV.htseq_fpkm) and data for normal ovarian tissues (dataset ID: dataset ID: gtex_RSEM_gene_fpkm) were extracted from The Cancer Genome Atlas (TCGA) and Genotype-Tissue Expression (GTEx), respectively. The gene symbols were re-annotated using Ensembl (https://uswest.ensembl.org/index.html) to select lncRNAs **[**14**]**, expressions of which come from 88 normal ovarian samples and 379 OC samples and were presented by log2 (fpkm+1). Then, ‘limma’ package (version 3.42.0) [15] was used to combine the two sets of lncRNAs into one with normalization. In total, 467 samples concerning 14,087 lncRNAs were filtered out, followed by removal of those genes with 0 expression.

### 2.2. DElncRNA identification

Using ‘stats’ package, version 3.6.2 **[**16**]**, principal component analysis (PCA) was performed to assess lncRNA expression distribution between OC and normal ovarian tissue. Using R language (limma package, R version 3.6.2), DElncRNAs between OC and normal ovarian tissue were determined according to the criteria set as | log2 (fold-change) | ≥ 2 and false discovery rate (FDR) < 0.05.

### 2.3. Prognostic lncRNA signature generation

Six OC samples were excluded due to the lack of survival data and clinical information. Running the ‘caret’ package, version 6.0–85 **[**17**]**, the DElncRNA profile was then randomly divided into a training cohort (n = 188) and a validation cohort (n = 185) according to the criteria that age, FIGO stage, and histological grade between the two groups were similar.

Data was divided into two groups, training cohort, where a survival analysis model was established by running ‘survival’ package (version 3.1–8) **[**18**]** and a separate validation cohort for model testing. First, the training cohort was used to perform analysis step by step as follows: 1. Kaplan–Meier (KM) method was used to assess the survival differences between the low and high expression groups of lncRNA. 2. Application of univariate Cox proportional hazards regression models to evaluate the association between DElncRNA expressions and overall survival (OS) where P-value < 0.05 was considered statistically significant. 3. Least absolute shrinkage and selection operator (LASSO) regression was performed to filter out the DElncRNAs according to the best value of lambda, thus eliminating overfitting of the model. 4. Multivariate Cox proportional hazards regression analysis was used to further select lncRNAs by a ‘step’ function, with results visualized as a forest plot. Finally, a prognostic prediction model including five DElncRNAs was constructed based on the regression coefficient-weighted lncRNA expression. We took the prognostic index as the risk score and a risk score formula, which has been well established in the literature **[**19**]**, was generated as follows:

$$Riskscore=\overset{n}{\underset{i=1}{\sum }}Expi*Coei$$

In the formula, N, Expi, and Coei represent the number of selected lncRNAs, expression of each lnc, and the multivariate Cox regression coefficient, respectively. A nomogram was constructed based on the lncRNA expression for predicting the survival of OC patients. We then extracted each corresponding lncRNA expression in the training cohort and substituted into the model, thereby generating every patient’s risk score, the median of which divided patients into low- and high-risk prognostic groups. Based on this, the receiver operating characteristic (ROC) curve (‘survivalROC’ packages, version 1.0.3) was used to assess the efficacies of the nomogram. Repetition was done in the validation cohort. Using DEnorm package (version 5.0.1), a free online calculator for the final nomogram was established and published in https://www.shinyapps.io/, in order to facilitate clinical use.

### 2.4. Construction of the lncRNA-miRNA-mRNA regulatory network

MiRNAs that potentially bind to lncRNAs were generated using DIANA tools **[**20**]**. Predictions of miRNA-targeting genes were made using three datasets, including miRDB **[**21**]**, miRTarBase **[**22**]**, and TargetScan **[**23**]**. Pearson analysis was performed to calculate the expression correlation between lncRNAs and miRNA-targeting genes. Target genes with | r | ≥ 0.3 were selected and Cytoscape (version 3.7.2) was used to construct the lncRNA-miRNA-mRNA regulatory networks.

### 2.5. Functional annotations and signaling pathway enrichment analysis

Gene Oncology (GO) and Kyoto Encyclopedia of Genes and Genomes (KEGG) pathway analysis of the risk signature were performed to analyze the target genes via the Metascape website **[**24**]**. GO results and KEGG pathways with FDR < 0.05 were selected and shown in charts.

### 2.6. Identification of candidate small molecule drugs

Connectivity Map (CMap) (version build 02) is a database exploring connections among small molecules and genes **[**25**]**. With its usage, candidate small-molecule drugs OC were predicted based on the rationale of targeting the ceRNA networks. The enrichment scores (ranging from −1 to 1) representing similarity were measured and drugs with negative connectivity were considered as potential therapeutics. To select drugs that are more likely to be effective, we set the enrichment scores < −0.85 and p-value < 0.001 as selection criteria. Tomographs of the candidate drugs were queried in the PubChem database.

### 2.7. Statistical analysis

All statistical tests were conducted using R programming language. Quantitative data are presented as the mean ± standard deviation. Statistical differences between the two groups were examined using the Wilcoxon test. P-value < 0.05 was considered statistically significant.

## 3. Results

In this study, we developed a prognostic model by using DElncRNAs to predict the survival of OC. Afterward, we established ceRNA regulatory networks for biological function, pathway exploration in addition to candidate drugs prediction. The results were as follows:

### 3.1. Identification of five prognostic DElncRNAs

**Figure 1.**
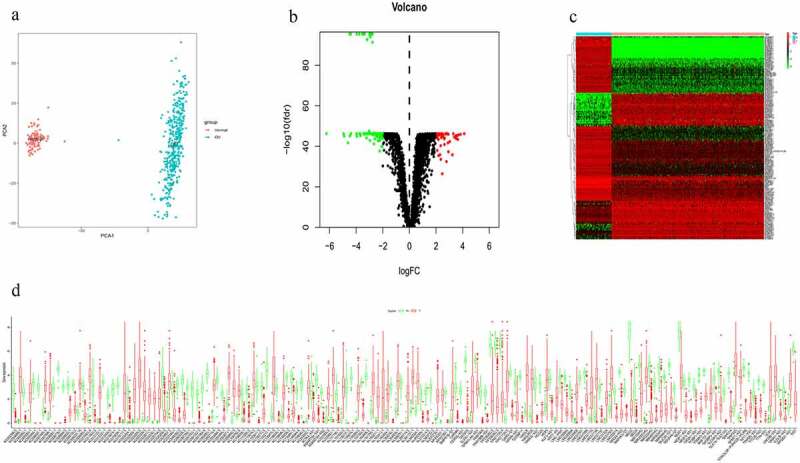
Identification of differentially expressed lncRNAs (DElncRNAs) for constructing the risk signature for OC. (a) Principal components analysis of lncRNAs between OC and normal ovarian tissue. (b) Volcano plot shows the distribution of DElncRNAs. Red and green dots represent the up-regulated and down-regulated DElncRNAs with | log2(fold-change) | ≥ 2, respectively (c) Heatmap exhibits the expression levels of the DElncRNAs. (d) Boxplot shows the detail of DElncRNAs. Red and green boxes indicate the lncRNA expression in OC and in normal ovarian tissue, respectively

**Figure 2.**
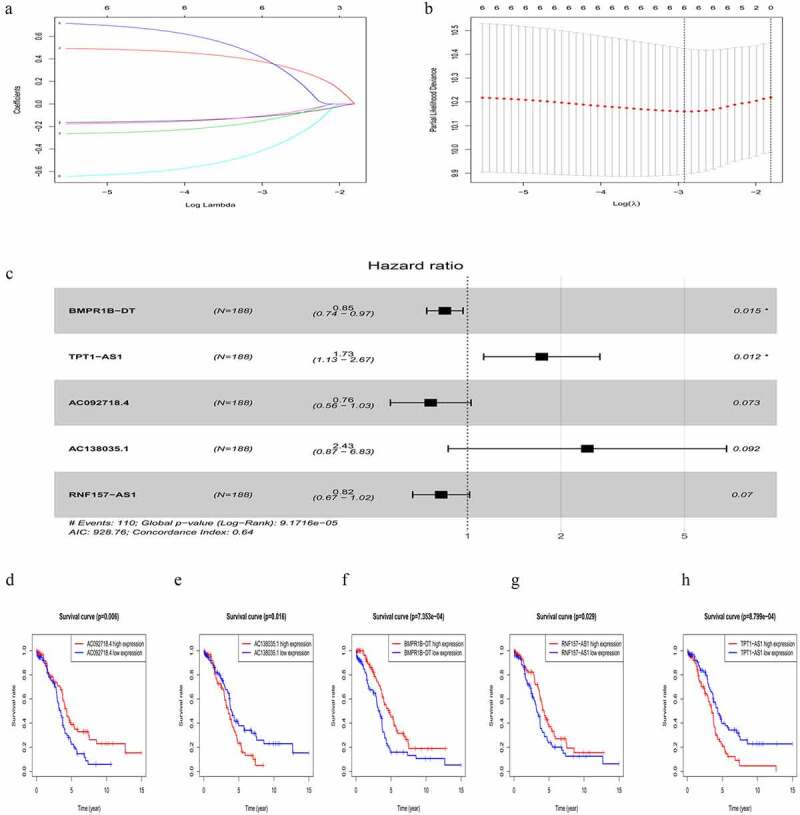
Differentially expressed lncRNA (DElncRNAs) for risk signature construction. (a) Log (Lambda) value of the 7 lncRNAs in the least absolute shrinkage and selection operator (LASSO) regression. (b) The most appropriate log (Lambda) value in the LASSO model. (c) Multivariate Cox regression analysis was performed and 5 lncRNAs *(AC092718.4, AC138035.1, BMPR1B-DT, RNF157-AS1*, and *TPT1-AS1)* were selected to construct the risk signature. (d-h) Kaplan–Meier analysis showed overall survival differences between low-risk and high-risk groups in *AC092718.4, AC138035.1, BMPR1B-DT, RNF157-AS1*, and *TPT1-AS1*, respectively

A total of 14,087 lncRNAs were included in this study. To reduce the dimensionality, PCA was performed to increase the interpretability while minimizing the information loss at the same time. As a result, the distribution of the lncRNAs between OC and normal ovarian samples was clearly shown (Figure 1a). To reflect the distinct characteristics of OC and normal ovarian tissue, 158 DElncRNAs between these two datasets were selected for further study (Figure 1b, 1c). Each lncRNA with median expression levels in OC and normal ovarian tissues was shown (Figure 1d). OC patients in the TCGA dataset with detailed clinical information (age, clinical stage, histologic grade, survival time, and status) were enrolled and randomly divided into a training cohort (n = 188) and a validation cohort (n = 185) (Table S1). To conﬁrm the prognostic value of these DElncRNAs, we performed a KM analysis in the training cohort and 15 potential prognostic DElncRNAs were screened out (Table S2), followed by the identification of 7 DElncRNAs that were correlated with OC prognosis (Table S3). To select appropriate parameters for constructing a risk signature, LASSO regression was used, six DElncRNAs were identiﬁed (*AC011603.2, AC092718.4, AC138035.1, BMPR1B-DT, RNF157-AS1*, and *TPT1-AS1*) (Figure 2a, b). Eventually, there were only ﬁve DElncRNAs (*AC092718.4, AC138035.1, BMPR1B-DT, RNF157-AS1*, and *TPT1-AS1*) left following multivariate Cox regression analysis. *BMPR1B-DT, AC092718.4,* and *RNF157-AS1* were regarded as protective factors (HR < 1), while *AC138035.1* and *TPT1-AS1* were deﬁned as risk factors (HR > 1) in OC (Figure 2c).

As shown in Figure 2d-H, higher levels of *AC138035.1* or *TPT1-AS1* exhibited poorer prognosis compared to those in the lower expression group. On the contrary, patients with higher expression of *BMPR1B-DT, AC092718.4,* and *RNF157-AS1* had relatively favorable prognosis. Based on both univariate and multivariate Cox regression analysis, ﬁve DElncRNAs, as prognostic biomarkers, were suggested for further analysis.

### 3.2. Establishment of an OC prognostic risk signature using the five lncRNAs

**Figure 3.**
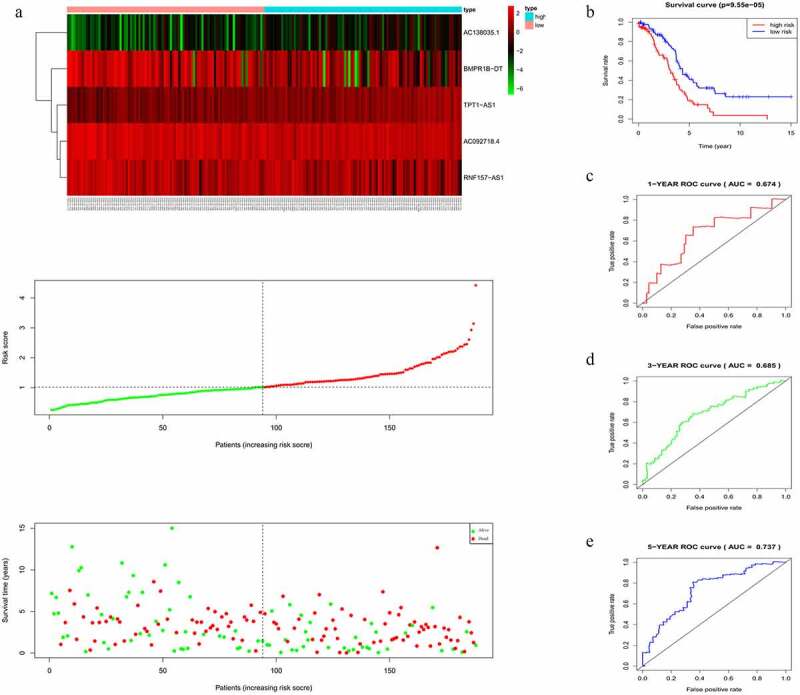
Characteristics of the five-lncRNA risk signature in the training cohort. (a) LncRNA expression profiles, risk score distributions and patient survival in the training cohort. (b) Survival curves for high-risk and low-risk groups decided by the risk signature in the training cohort. (c-e) ROC of the five-lncRNA risk signature in predicting the 1-, 3-, and 5-year survival in the training cohort

The risk score of each patient in the training cohort, calculated using the formula mentioned in the Materials and Methods 2.3, is as follows: risk score = (−0.2735 × expression level of *AC092718.4*) + (0.8891 × expression level of *AC138035.1*) + (−0.1682 × expression level of *BMPR1B-DT*) + (−0.1957 × expression level of *RNF157-AS1*) + (0.5509 × expression level of *TPT1-AS1*). Accordingly, patients were then divided into low – and high-risk groups based on the median risk score. As shown in Figure 3a, more deaths were seen with risk score rising. Interestingly, with the increasing in risk score, the expression levels of *BMPR1B-DT, AC092718.4,* and *RNF157-AS1* were decreased, whereas levels of *AC138035.1* and *TPT1-AS1* were upregulated. As the KM curve suggested, high-risk group tended to develop poorer prognoses (Figure 3b). The ROC curve used to assess the efficacy of survival prediction in OC patients showed that the areas under curve (AUC) for the 1-, 3-, and 5-year survivals were 0.674, 0.685, and 0.737, respectively (Figure 3c-e), indicating good predictive efficacy of the risk signature.

### 3.3. Validation of the five-lncRNA risk signature

**Figure 4.**
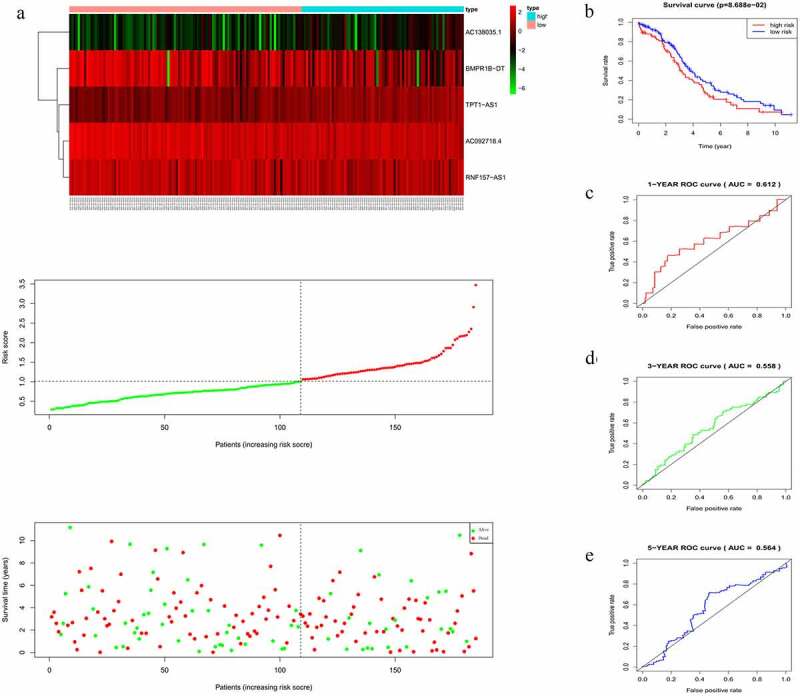
Evaluating the efficacy of the five-lncRNA risk signature in the validation cohort. (a) LncRNA expression profiles, risk score distributions and patient survival in the validation cohort. (b) Survival curves for high-risk and low-risk groups decided by the risk signature in the validation cohort. (c-e) ROC of the five-lncRNA risk signature in predicting the 1-, 3-, and 5-year survival in the validation cohort

In accordance with the results in the training cohort, down-regulation of *BMPR1B-DT, AC092718.4,* and *RNF157-AS1*, and up-regulation of the other two lncRNAs in the validation cohort corresponded to an increase in number of deaths, as reflected by increased risk scores (Figure 4a). KM curves also showed that the high-risk group had poorer prognoses (Figure 4b). AUCs for 1-, 3-, and 5-year survival were 0.612, 0.558, and 0.564, respectively (Figure 4c-e), thus confirming the good efficacy for survival prediction.

### 3.4. Construction of a nomogram based on the risk signature

To confirm the prognostic value of the risk signature, univariate and multivariate Cox regression analyses were conducted in the training cohort. It was observed that the risk signature of the ﬁve lncRNAs was independently associated with overall survival of OC (Figure S1A, S1B). Considering the potential clinical relevance, the prognostic values of age, histological grade, and FIGO stage were also evaluated. However, among these OC samples, factors such as age, clinical stage, and histologic grade were of no survival-prognostic value since their p-values were larger than 0.05. Thus, a nomogram was constructed using only the five lncRNAs. As the nomogram showed, the 1-, 3-, and 5-year survival incidences could quickly be determined according to the total points, by summing the points in each item (Figure S1C). An online software for the survival nomogram was utilized for easier clinical use by DEnorm package: https://linnuanqq.shinyapps.io/dynnomapp2/?_ga=2.1208497.1499237141.1594603552-159983587.1588641469.

### 3.5. LncRNA-miRNA-mRNA regulatory networks

**Figure 5.**
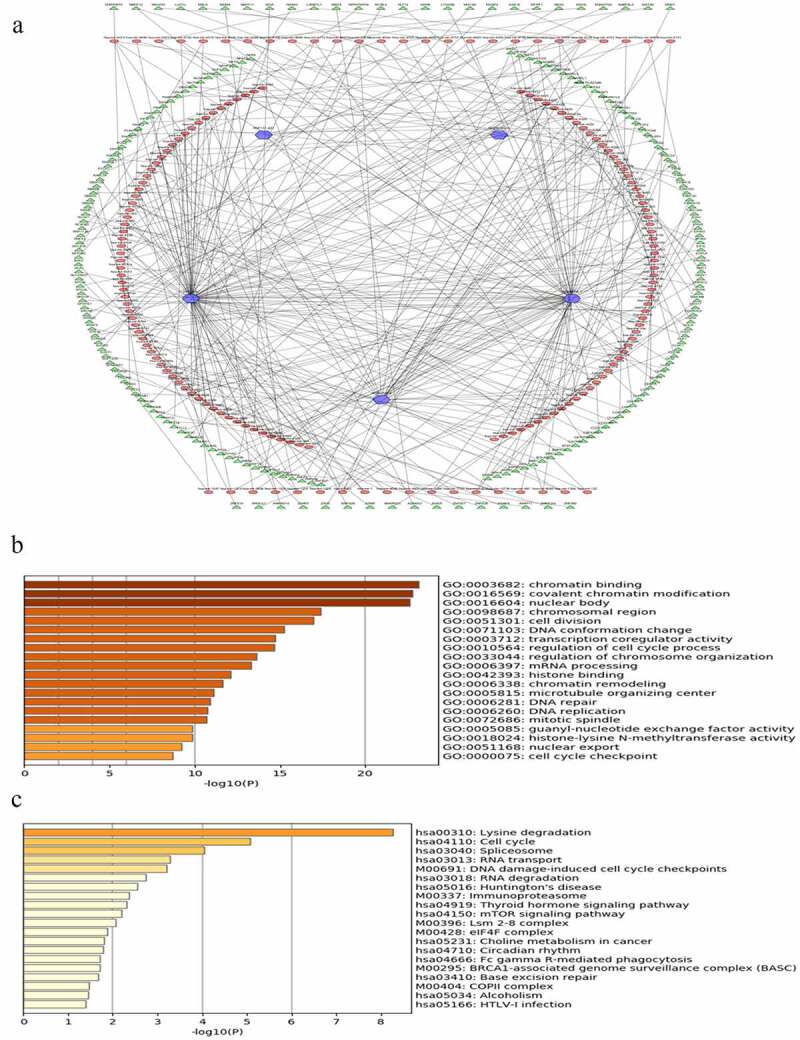
Construction of lncRNA-miRNA-mRNA regulatory networks with functional annotations and signaling pathways. (a) LncRNAs as well as their potential binding miRNAs and target genes with | r | ≥ 0.4 related to the five lncRNAs were used to construct the lncRNA-miRNA-mRNA regulatory networks. However, due to the huge number of genes in the networks, only mRNAs with | r | ≥ 0.4 are visualized here. Blue hexagons represent lncRNAs, which are located at the cores of the networks. Red ellipses and green triangles stand for miRNAs and mRNAs, respectively. Gene oncology (b) and KEGG pathway (c) analyses were performed based on the target genes of the networks via Metascape

LncRNAs can regulate gene expression by functioning as miRNA sponges and rescuing miRNA-targeted genes via ceRNA networks *[26]*. MiRNAs that could potentially bind to the five lncRNAs were identified and listed in Table S4. The predicted mRNAs that were common in at least two of the databases used (miRDB, miRTarBase, and TargetScan) and those with | r | ≥ 0.3 were selected as potential target genes, listed in Table S5. Finally, lncRNA-miRNA-mRNA regulatory networks were established based on 5 lncRNAs, 531 miRNAs, and 1639 mRNAs. However, due to the huge number of genes in the network, only mRNAs with | r | ≥ 0.4 were visualized by Cytoscape (Figure 5a).

### 3.6. Functional annotations and signaling pathway enrichments

To further investigate the functions of the prognostic signature, the biological processes and pathways, based on the constructed ceRNA networks, were explored. The signature was closely related to cancer-associated biological processes, such as chromatin binding, covalent chromatin modification, and regulation of cell cycle process (Figure 5b, p-value < 0.05). Similarly, several KEGG pathways including cell cycle, DNA damage-induced cell cycle checkpoints, the mTOR signaling pathway, and the ovarian-specific BRCA1-associated genome surveillance complex (BASC) were identiﬁed (Figure 5c, p-value < 0.05). Taken together, these results suggest that the risk signature is correlated with the behavior of OC.

### 3.7. Five candidate small molecule drugs

A total of 1639 genes from the regulatory networks were analyzed in CMap, and five candidate small molecules with potential therapeutic value were predicted (Table 1). Thioridazine, trifluoperazine, loperamide, LY294002, and puromycin were all negatively correlated with the upregulated genes, and their tomographies were shown (Figure S2).

**Table 1.** The top 10 OC-related small molecules with highly significant correlations in the results of CMap

rank	cmap name	mean	n	enrichment	p-value
1	puromycin	−0.86	4	−0.982	0
2	thioridazine	−0.455	20	−0.529	0
3	LY-294,002	−0.435	61	−0.501	0
4	loperamide	−0.654	6	−0.869	0.00002
5	trifluoperazine	−0.313	16	−0.495	0.00034
6	bumetanide	0.458	4	0.868	0.0004
7	atractyloside	0.347	5	0.819	0.00042
8	5,230,742	−0.814	2	−0.983	0.00058
9	15-delta prostaglandin J2	−0.392	15	−0.482	0.00108
10	cefamandole	0.517	4	0.834	0.00115

## 4. Discussion

The prognosis of OC remains poor, and there are few effective prognostic biomarkers or models for improving the clinical outcomes of OC patients. In the present study, we identify five key lncRNAs (*TPT1-AS1, RNF157-AS1, BMPR1B-DT, AC092718.4,* and *AC138035.1*) with a constructed risk signature for predicting survival of OC.

*TPT1-AS1 (TPT1 antisense RNA 1)* is located on chromosome 13 and has been reported to promote proliferation and metastasis in a variety of cancers [27,28]. Interestingly, our finding of *TPT1-AS1* as a potential therapeutic target for OC is corroborated by the study of Wu et al., who recently showed that TPT1-AS1 induces OC tumor growth and metastasis [29]. Limited research on *RNF157-AS1 (RNF157 antisense RNA 1)* has been published so far, with only a microarray analysis suggesting its up-regulation in OC, followed by quantitative real-time PCR validation [30]. In addition to this previous finding, our results also suggest that *RNF157-AS1* is a positive predictor for survival of OC patients. *BMPR1B-DT* is a divergent transcript of its neighboring protein-coding gene *BMPR1B (ALK6)*. However, the role of *BMPR1B-DT* has not been elaborated. Transcription of the most divergent lncRNAs makes the adjacent gene locus more accessible or alternatively, inaccessible by competing for mRNA gene promoters [31,32]. Expression of *BMPR1B* is highest in adult ovaries, and is the major gene identified for reproductive traits including folliculogenesis. While it was reported that OC patients with *BMPR1B* expression have an unfavorable prognosis [33], our results indicate that the lncRNA *BMPR1B-DT* predicts longer survival. Taken together, it is conceivable that lncRNA *BMPR1B-DT* is a competitor for the *BMPR1B* mRNA promoter. Despite emerging studies on the biological functions of lncRNAs, the role of the remaining two lncRNAs (*AC092718.4, AC138035.1*) in OC progression is not documented. Here, for the first time, we show that *AC092718.4* has an HR < 1, indicating it is a positive predictor for OC. In contrast, a higher expression of *AC138035.1* suggests an unfavorable prognosis in OC. Therefore, according to our study, all five lncRNAs can serve as candidate prognostic markers and therapeutic targets of OC, although further studies are warranted.

Emerging evidence suggests that lncRNAs play a vital role in a variety of physiological functions and disease progression, including cancer [12,13]. Importantly, some lncRNAs compete for specific miRNA target sites, thus, regulating gene expression via acting as ceRNAs [7]. In this study, we constructed lncRNA-miRNA-mRNA regulatory networks to show the relationships of the lncRNAs *(AC092718.4, AC138035.1, BMPR1B-DT, RNF157-AS1*, and *TPT1-AS1)* along with their binding miRNAs and target genes. To explore the biological functions and signaling pathways of the risk signature, further gene enrichment analysis was performed, with results indicating that the ceRNA networks can play a role in cancer-related biological processes, such as regulation of cell cycle, chromatin binding, modification and remodeling, mTOR signaling pathway, and the ovarian-cancer specific BASC.

Currently, chemotherapeutic drugs including platinum, carboplatin, and paclitaxel are widely used among OC patients, with challenges posed by drug resistance and cancer recurrence. Molecular targets and pathways currently under investigation for drug development in ovarian cancers include, cell cycle inhibitors, mTOR inhibitors, and PI3K inhibitors. A recent study found that cisatracurium can inhibit the progression of OC cells by upregulating lncRNA-p21 activated by p53 inhibiting miR-181b expression, indicating that drugs targeting ceRNA networks are indeed promising in treating OC [34]. In our study, we found five small-molecule drugs with potential therapeutic efficacy based on OC RNA-seq using bioinformatics analysis, thus guiding the direction for future drug exploration. Thioridazine, a potent anti-psychotic and anti-anxiety agent, was specifically demonstrated to act against OC, possibly by targeting the mTOR pathway [35]. Trifluoperazine, is another widely used clinical antipsychotic agent that reportedly regulates cell cycle progression and has been shown to potentiate cisplatin toxicity in ovarian carcinoma cells [36,37]. As a classical antidiarrhea agent, loperamide is being increasingly recognized as a tumor suppressor in several cancer cells, including ovarian cancer, via inducing G2/M-phase cell cycle arrest [38,39]. LY294002, a potent PI3-K inhibitor, has been shown to inhibit tumor growth and ascites formation in OC [40]. Puromycin is a tRNA mimic traditionally viewed as an antibiotic [41]. Unlike the other four small molecules, its efficacy has never been reported in OC. However, puromycin induced apoptosis in breast cancer cells mediated by *insulin-like growth factor 1 (IGF-I)* [42]. Considering that the involvement of IGF-I in ovarian tumorigenesis has been supported by IGF-I-targeting strategies and ongoing clinical trials [43], a therapeutic potential of puromycin in OC can be anticipated. Nonetheless, further investigation is required in xenograft models and in clinical trials to gain more insight into these five potential therapeutic drugs for clinical application.

Zhou M et al., recently identified 10 prognostic lncRNAs and the associated ceRNA in ovarian cancer using the TCGA database [44]. However, it is not surprising that there is no overlap between the 10 lncRNAs identified by their analyses and the 5 lncRNAs proposed by us, since in the current study, it is the aberrantly expressed lncRNA between OC and normal ovarian tissue that were identified. An important advantage of this study is the use of transcriptome data from the GTEx Project [45], which allows accessibility to a much larger source of ovarian tissue data, while minimizing the measurement bias compared to data extraction from different GEO datasets. In addition to that, we made significant improvements in survival prediction in OC patients. First, LASSO regression was used to identify lncRNA in order to avoid overfitting of the model. Secondly, we constructed lncRNA-miRNA-mRNA regulatory networks and described the underlying functions of the risk signature in OC based on highly correlated genes. Thirdly, we validated the established risk signature in a validation group, thereby testing its accuracy in predicting survival. Nonetheless, we recognize that our study has some limitations. First, the data of ovarian cancer samples with clinicopathological information included are still limited. Additionally, the histologic grade and FIGO stage were of no predictive value for OC survival, probably due to the statistically increased false-negative rate, when samples in some sub-groups were too small to be followed by sub-staging and sub-grading [46]. Nevertheless, our risk score has good performance in predicting the 1-, 3-, and 5-year survival of the validation cohort, indicating that molecular features may be more stable in prognosis. Moreover, some other confounding factors of survival, such as surgical resection and chemotherapy, were not available in the datasets and thus not taken into account. Finally, the validation cohort was based on 185 retrospective cases from TCGA, so we may replicate our study in larger cohorts with comprehensive clinical information to validate the five-lncRNA signature in future studies.

## 5. Conclusion

Our study complements available genomic-based studies, identifies lncRNA biomarkers, establishes a five-lncRNA signature for survival prediction, and constructs ceRNA networks for exploration of potentially more selective drugs for OC.

## Supplementary Material

Supplemental Material
