# Supplementary material for: Identification and validation of a five-lncRNA signature for predicting survival with targeted drug candidates in ovarian cancer
Source: Bioengineered. 2021 Jul 5;12(1):3263–74. doi: 10.1080/21655979.2021.1946632 (PMC8806566; doi:10.1080/21655979.2021.1946632)
Supplement: Supplemental Material [file KBIE_A_1946632_SM7043.zip › supplementary/Table S1.docx]

**Table S1. Clinicopathological characteristics of samples in the training cohort and the validation cohort**

| **clinicopathological** | | | | **no. of sample** | |
| --- | --- | --- | --- | --- | --- |
| **characteristics** |  |  |  |  |  |
|  |  | **training cohort(n=188)** | | | **validation cohort(n=185)** |
| **clinical_stage** | |  |  |  |  |
| stage I |  | 0 |  |  | 1 |
| stage II |  | 8 |  |  | 13 |
| stage III | | 147 |  |  | 144 |
| stage IV |  | 31 |  |  | 26 |
| UNKNOWN |  | 2 |  |  | 1 |
| **WHO grade** | |  |  |  |  |
| I |  | 1 |  |  | 0 |
| II |  | 21 |  |  | 20 |
| III |  | 161 |  |  | 159 |
| IV |  | 0 |  |  | 1 |
| B |  | 1 |  |  | 1 |
| X |  | 4 |  |  | 2 |
| UNKNOWN |  | 0 |  |  | 2 |
| **Age** |  |  |  |  |  |
| average value | | 36-85 |  |  | 30-87 |
| range |  | 59.57 |  |  | 59.42 |
