# Supplementary material for: Identification and validation of a five-lncRNA signature for predicting survival with targeted drug candidates in ovarian cancer
Source: Bioengineered. 2021 Jul 5;12(1):3263–74. doi: 10.1080/21655979.2021.1946632 (PMC8806566; doi:10.1080/21655979.2021.1946632)
Supplement: Supplemental Material [file KBIE_A_1946632_SM7043.zip › supplementary/Table S2.docx]

**Table S2. The potential prognostic genes by K-M analyzing**

| **gene** | **P value** |
| --- | --- |
| BMPR1B-DT | 0.000735 |
| TPT1-AS1 | 0.00088 |
| AC092718.4 | 0.0062 |
| GARS-DT | 0.007178 |
| AC016026.1 | 0.011655 |
| AL157392.3 | 0.012217 |
| AL390719.2 | 0.014099 |
| AC138035.1 | 0.016304 |
| AC007405.3 | 0.018771 |
| AC011603.2 | 0.02646 |
| AC110285.2 | 0.027035 |
| RNF157-AS1 | 0.028879 |
| MAP4K3-DT | 0.030975 |
| STAG3L5P-PVRIG2P-PILRB | 0.033515 |
| NDUFA6-DT | 0.03757 |
