# Supplementary material for: Identification and validation of a five-lncRNA signature for predicting survival with targeted drug candidates in ovarian cancer
Source: Bioengineered. 2021 Jul 5;12(1):3263–74. doi: 10.1080/21655979.2021.1946632 (PMC8806566; doi:10.1080/21655979.2021.1946632)
Supplement: Supplemental Material [file KBIE_A_1946632_SM7043.zip › supplementary/Table S3.docx]

**Table S3. The potential prognostic genes by univariate Cox analyzing**

| **id** | **HR** | **HR.95L** | **HR.95H** | **pvalue** |
| --- | --- | --- | --- | --- |
| TPT1-AS1 | 2.004561 | 1.295824 | 3.100934 | 0.001783 |
| BMPR1B-DT | 0.833152 | 0.729713 | 0.951255 | 0.006959 |
| RNF157-AS1 | 0.785662 | 0.639569 | 0.965126 | 0.021554 |
| AC138035.1 | 3.089936 | 1.178446 | 8.101946 | 0.021801 |
| AL157392.3 | 1.955334 | 1.099162 | 3.478406 | 0.022508 |
| AC092718.4 | 0.723079 | 0.545202 | 0.958988 | 0.024408 |
| AC011603.2 | 0.319193 | 0.109547 | 0.930044 | 0.03636 |
