# Supplementary material for: Identification and validation of a five-lncRNA signature for predicting survival with targeted drug candidates in ovarian cancer
Source: Bioengineered. 2021 Jul 5;12(1):3263–74. doi: 10.1080/21655979.2021.1946632 (PMC8806566; doi:10.1080/21655979.2021.1946632)
Supplement: Supplemental Material [file KBIE_A_1946632_SM7043.zip › supplementary/Table S4.docx]

**Table S4. Potential miRNAs binding to the five lncRNAs**

| **lncRNA** | **miRNA** |
| --- | --- |
| AC092718.4 | hsa-mir-93-5p,hsa-mir-887-5p,hsa-mir-877-5p,hsa-mir-8086,hsa-mir-8085,hsa-mir-8082,hsa-mir-7973,hsa-mir-7856-5p,hsa-mir-764,hsa-mir-7160-5p,hsa-mir-7155-3p,hsa-mir-6878-5p,hsa-mir-6874-5p,hsa-mir-6856-5p,hsa-mir-6832-5p,hsa-mir-6815-3p,hsa-mir-6804-5p,hsa-mir-6797-5p,hsa-mir-6794-3p,hsa-mir-6788-5p,hsa-mir-6780a-5p,hsa-mir-6779-5p,hsa-mir-6758-5p,hsa-mir-6755-5p,hsa-mir-6749-3p,hsa-mir-6747-3p,hsa-mir-6738-5p,hsa-mir-6731-5p,hsa-mir-6718-5p,hsa-mir-6514-5p,hsa-mir-6513-5p,hsa-mir-6506-5p,hsa-mir-645,hsa-mir-619-5p,hsa-mir-6133,hsa-mir-607,hsa-mir-590-3p,hsa-mir-581,hsa-mir-5701,hsa-mir-5690,hsa-mir-548c-3p,hsa-mir-526b-3p,hsa-mir-5194,hsa-mir-512-3p,hsa-mir-504-3p,hsa-mir-5007-5p,hsa-mir-5002-5p,hsa-mir-498,hsa-mir-495-5p,hsa-mir-488-3p,hsa-mir-4786-3p,hsa-mir-4785,hsa-mir-4784,hsa-mir-4779,hsa-mir-4775,hsa-mir-4766-5p,hsa-mir-4761-5p,hsa-mir-4757-5p,hsa-mir-4731-5p,hsa-mir-4729,hsa-mir-4709-3p,hsa-mir-4696,hsa-mir-4689,hsa-mir-4662b,hsa-mir-4647,hsa-mir-4639-5p,hsa-mir-4534,hsa-mir-4524b-3p,hsa-mir-4524a-3p,hsa-mir-450a-1-3p,hsa-mir-4474-5p,hsa-mir-4469,hsa-mir-4451,hsa-mir-4430,hsa-mir-4429,hsa-mir-4426,hsa-mir-4309,hsa-mir-4299,hsa-mir-4294,hsa-mir-4284,hsa-mir-4282,hsa-mir-411-5p,hsa-mir-3915,hsa-mir-3913-5p,hsa-mir-382-5p,hsa-mir-371a-5p,hsa-mir-3689c,hsa-mir-3689b-3p,hsa-mir-3689a-3p,hsa-mir-367-3p,hsa-mir-3664-5p,hsa-mir-3652,hsa-mir-363-3p,hsa-mir-335-3p,hsa-mir-32-3p,hsa-mir-320d,hsa-mir-320c,hsa-mir-320b,hsa-mir-320a,hsa-mir-3198,hsa-mir-3162-5p,hsa-mir-3150b-3p,hsa-mir-3136-3p,hsa-mir-3135b,hsa-mir-3132,hsa-mir-3125,hsa-mir-3122,hsa-mir-30c-2-3p,hsa-mir-30c-1-3p,hsa-mir-30b-3p,hsa-mir-26b-5p,hsa-mir-26a-5p,hsa-mir-23a-5p,hsa-mir-219b-3p,hsa-mir-20b-5p,hsa-mir-20a-5p,hsa-mir-208a-5p,hsa-mir-2052,hsa-mir-204-3p,hsa-mir-1976,hsa-mir-197-5p,hsa-mir-197-3p,hsa-mir-193b-5p,hsa-mir-1914-3p,hsa-mir-17-5p,hsa-mir-146a-3p,hsa-mir-143-5p,hsa-mir-142-3p,hsa-mir-138-5p,hsa-mir-136-5p,hsa-mir-135b-3p,hsa-mir-1305,hsa-mir-1304-3p,hsa-mir-1303,hsa-mir-1297,hsa-mir-129-5p,hsa-mir-1289,hsa-mir-1273h-5p,hsa-mir-1249-5p,hsa-mir-122-5p,hsa-mir-10b-3p,hsa-mir-106b-5p,hsa-mir-106a-5p,hsa-mir-106a-3p |
| AC138035.1 | hsa-mir-98-5p,hsa-mir-940,hsa-mir-937-5p,hsa-mir-93-3p,hsa-mir-92a-1-5p,hsa-mir-891a-3p,hsa-mir-887-5p,hsa-mir-885-5p,hsa-mir-877-5p,hsa-mir-875-3p,hsa-mir-8085,hsa-mir-8082,hsa-mir-8081,hsa-mir-8077,hsa-mir-8052,hsa-mir-7977,hsa-mir-7974,hsa-mir-7847-3p,hsa-mir-769-5p,hsa-mir-767-3p,hsa-mir-766-3p,hsa-mir-759,hsa-mir-758-3p,hsa-mir-744-3p,hsa-mir-7160-5p,hsa-mir-7158-3p,hsa-mir-7154-5p,hsa-mir-7152-5p,hsa-mir-7150,hsa-mir-7110-3p,hsa-mir-7107-3p,hsa-mir-7106-5p,hsa-mir-6895-3p,hsa-mir-6894-5p,hsa-mir-6893-5p,hsa-mir-6890-3p,hsa-mir-6884-5p,hsa-mir-6883-5p,hsa-mir-6880-5p,hsa-mir-6879-3p,hsa-mir-6875-3p,hsa-mir-6873-5p,hsa-mir-6873-3p,hsa-mir-6871-3p,hsa-mir-6866-5p,hsa-mir-6865-5p,hsa-mir-6860,hsa-mir-6858-3p,hsa-mir-6849-3p,hsa-mir-6848-3p,hsa-mir-6847-5p,hsa-mir-6847-3p,hsa-mir-6843-3p,hsa-mir-6839-5p,hsa-mir-6834-5p,hsa-mir-6832-5p,hsa-mir-6830-3p,hsa-mir-6823-3p,hsa-mir-6817-3p,hsa-mir-6815-5p,hsa-mir-6811-3p,hsa-mir-6809-5p,hsa-mir-6808-5p,hsa-mir-6807-5p,hsa-mir-6802-3p,hsa-mir-6799-5p,hsa-mir-6795-3p,hsa-mir-6790-5p,hsa-mir-6788-5p,hsa-mir-6787-3p,hsa-mir-6785-5p,hsa-mir-6783-3p,hsa-mir-6780a-5p,hsa-mir-6779-5p,hsa-mir-6778-5p,hsa-mir-6776-5p,hsa-mir-6775-3p,hsa-mir-6773-5p,hsa-mir-6767-3p,hsa-mir-6765-5p,hsa-mir-6762-3p,hsa-mir-6758-5p,hsa-mir-6753-3p,hsa-mir-6748-3p,hsa-mir-6744-3p,hsa-mir-6742-3p,hsa-mir-6736-5p,hsa-mir-6735-3p,hsa-mir-6731-5p,hsa-mir-6730-5p,hsa-mir-6724-5p,hsa-mir-6720-5p,hsa-mir-6718-5p,hsa-mir-663b,hsa-mir-662,hsa-mir-661,hsa-mir-657,hsa-mir-656-5p,hsa-mir-655-5p,hsa-mir-6515-3p,hsa-mir-6513-5p,hsa-mir-6513-3p,hsa-mir-6512-3p,hsa-mir-6511a-5p,hsa-mir-6503-5p,hsa-mir-6500-5p,hsa-mir-650,hsa-mir-627-5p,hsa-mir-620,hsa-mir-619-5p,hsa-mir-616-3p,hsa-mir-6134,hsa-mir-6133,hsa-mir-6128,hsa-mir-612,hsa-mir-6089,hsa-mir-6087,hsa-mir-6086,hsa-mir-604,hsa-mir-596,hsa-mir-585-5p,hsa-mir-585-3p,hsa-mir-583,hsa-mir-575,hsa-mir-5708,hsa-mir-5704,hsa-mir-5703,hsa-mir-5702,hsa-mir-5690,hsa-mir-5682,hsa-mir-5681a,hsa-mir-563,hsa-mir-5591-5p,hsa-mir-5589-5p,hsa-mir-5588-5p,hsa-mir-5586-5p,hsa-mir-558,hsa-mir-5572,hsa-mir-556-5p,hsa-mir-542-3p,hsa-mir-539-5p,hsa-mir-519c-3p,hsa-mir-519b-3p,hsa-mir-519a-3p,hsa-mir-5195-5p,hsa-mir-518c-5p,hsa-mir-5189-5p,hsa-mir-513b-5p,hsa-mir-5089-5p,hsa-mir-508-5p,hsa-mir-505-5p,hsa-mir-504-5p,hsa-mir-5008-5p,hsa-mir-494-5p,hsa-mir-489-3p,hsa-mir-488-3p,hsa-mir-486-5p,hsa-mir-485-5p,hsa-mir-4799-3p,hsa-mir-4793-5p,hsa-mir-4786-5p,hsa-mir-4772-3p,hsa-mir-4770,hsa-mir-4768-3p,hsa-mir-4765,hsa-mir-4758-5p,hsa-mir-4744,hsa-mir-4736,hsa-mir-4733-5p,hsa-mir-4732-5p,hsa-mir-4731-5p,hsa-mir-4728-5p,hsa-mir-4727-3p,hsa-mir-4725-5p,hsa-mir-4724-5p,hsa-mir-4722-5p,hsa-mir-4720-5p,hsa-mir-4717-5p,hsa-mir-4715-3p,hsa-mir-4709-5p,hsa-mir-4704-3p,hsa-mir-4691-3p,hsa-mir-4687-3p,hsa-mir-4676-5p,hsa-mir-4668-3p,hsa-mir-4659b-5p,hsa-mir-4658,hsa-mir-4649-3p,hsa-mir-4644,hsa-mir-4638-3p,hsa-mir-4633-3p,hsa-mir-4539,hsa-mir-4538,hsa-mir-4534,hsa-mir-4531,hsa-mir-4530,hsa-mir-4529-5p,hsa-mir-4527,hsa-mir-4524a-3p,hsa-mir-450a-1-3p,hsa-mir-4505,hsa-mir-4504,hsa-mir-4503,hsa-mir-4500,hsa-mir-449c-5p,hsa-mir-449b-5p,hsa-mir-4487,hsa-mir-4486,hsa-mir-4478,hsa-mir-4474-5p,hsa-mir-4472,hsa-mir-4468,hsa-mir-4460,hsa-mir-4458,hsa-mir-4453,hsa-mir-4451,hsa-mir-4443,hsa-mir-4436a,hsa-mir-4435,hsa-mir-4434,hsa-mir-4433a-5p,hsa-mir-4430,hsa-mir-4425,hsa-mir-4419b,hsa-mir-4419a,hsa-mir-4329,hsa-mir-4326,hsa-mir-4324,hsa-mir-432-3p,hsa-mir-4316,hsa-mir-4306,hsa-mir-4300,hsa-mir-4293,hsa-mir-4289,hsa-mir-4267,hsa-mir-4264,hsa-mir-4259,hsa-mir-4257,hsa-mir-4254,hsa-mir-4253,hsa-mir-4252,hsa-mir-4251,hsa-mir-421,hsa-mir-410-5p,hsa-mir-3975,hsa-mir-3945,hsa-mir-3942-3p,hsa-mir-3938,hsa-mir-3935,hsa-mir-3929,hsa-mir-3925-3p,hsa-mir-3919,hsa-mir-3913-5p,hsa-mir-380-5p,hsa-mir-379-5p,hsa-mir-378j,hsa-mir-378g,hsa-mir-378a-5p,hsa-mir-377-5p,hsa-mir-376a-2-5p,hsa-mir-3690,hsa-mir-3689d,hsa-mir-3689c,hsa-mir-3689b-3p,hsa-mir-3689a-3p,hsa-mir-3688-5p,hsa-mir-3668,hsa-mir-3665,hsa-mir-3655,hsa-mir-3612,hsa-mir-34c-5p,hsa-mir-34b-5p,hsa-mir-33b-5p,hsa-mir-33a-5p,hsa-mir-338-3p,hsa-mir-323b-5p,hsa-mir-3199,hsa-mir-3192-5p,hsa-mir-3190-5p,hsa-mir-3188,hsa-mir-3187-5p,hsa-mir-3165,hsa-mir-3160-3p,hsa-mir-31-5p,hsa-mir-3159,hsa-mir-3158-5p,hsa-mir-3157-5p,hsa-mir-3147,hsa-mir-3145-5p,hsa-mir-3143,hsa-mir-3139,hsa-mir-3137,hsa-mir-3122,hsa-mir-3116,hsa-mir-30e-3p,hsa-mir-30d-3p,hsa-mir-30c-2-3p,hsa-mir-30c-1-3p,hsa-mir-30b-3p,hsa-mir-30a-3p,hsa-mir-3074-5p,hsa-mir-302f,hsa-mir-29c-3p,hsa-mir-29b-3p,hsa-mir-29a-3p,hsa-mir-2861,hsa-mir-2682-5p,hsa-mir-2467-5p,hsa-mir-2467-3p,hsa-mir-24-3p,hsa-mir-2276-5p,hsa-mir-219b-3p,hsa-mir-214-5p,hsa-mir-20b-3p,hsa-mir-204-3p,hsa-mir-203b-5p,hsa-mir-1914-5p,hsa-mir-1910-3p,hsa-mir-18b-5p,hsa-mir-18a-5p,hsa-mir-185-5p,hsa-mir-1827,hsa-mir-181a-2-3p,hsa-mir-17-3p,hsa-mir-15a-3p,hsa-mir-153-5p,hsa-mir-149-3p,hsa-mir-148b-5p,hsa-mir-148a-5p,hsa-mir-143-3p,hsa-mir-140-5p,hsa-mir-139-3p,hsa-mir-136-5p,hsa-mir-133b,hsa-mir-130b-5p,hsa-mir-1304-5p,hsa-mir-1299,hsa-mir-1295b-3p,hsa-mir-1294,hsa-mir-1291,hsa-mir-1286,hsa-mir-1285-3p,hsa-mir-1273h-5p,hsa-mir-1273g-3p,hsa-mir-1273f,hsa-mir-1273e,hsa-mir-1270,hsa-mir-1268b,hsa-mir-1268a,hsa-mir-1266-3p,hsa-mir-1260b,hsa-mir-125b-2-3p,hsa-mir-1254,hsa-mir-1238-5p,hsa-mir-1236-5p,hsa-mir-1236-3p,hsa-mir-1233-5p,hsa-mir-1227-3p,hsa-mir-1225-3p,hsa-mir-1224-5p,hsa-mir-1207-5p,hsa-mir-1200,hsa-mir-1199-5p,hsa-mir-100-3p,hsa-let-7i-5p,hsa-let-7g-5p,hsa-let-7f-5p,hsa-let-7e-5p,hsa-let-7c-5p,hsa-let-7a-5p |
| BMPR1B-DT | hsa-mir-891a-5p,hsa-mir-8485,hsa-mir-7853-5p,hsa-mir-7849-3p,hsa-mir-7154-5p,hsa-mir-7152-5p,hsa-mir-6895-5p,hsa-mir-6893-3p,hsa-mir-6853-3p,hsa-mir-6836-5p,hsa-mir-6811-5p,hsa-mir-6754-3p,hsa-mir-6511b-5p,hsa-mir-6502-3p,hsa-mir-6132,hsa-mir-603,hsa-mir-592,hsa-mir-580-5p,hsa-mir-548an,hsa-mir-541-5p,hsa-mir-5092,hsa-mir-4770,hsa-mir-4761-5p,hsa-mir-4760-3p,hsa-mir-4731-3p,hsa-mir-4504,hsa-mir-412-3p,hsa-mir-3918,hsa-mir-371a-5p,hsa-mir-3650,hsa-mir-362-3p,hsa-mir-329-3p,hsa-mir-3074-5p,hsa-mir-1825,hsa-mir-17-3p,hsa-mir-153-5p,hsa-mir-143-3p,hsa-mir-132-5p,hsa-mir-105-5p |
| RNF157-AS1 | hsa-mir-942-5p,hsa-mir-922,hsa-mir-8485,hsa-mir-764,hsa-mir-7152-3p,hsa-mir-7110-3p,hsa-mir-708-5p,hsa-mir-6888-5p,hsa-mir-6885-3p,hsa-mir-6881-5p,hsa-mir-6873-5p,hsa-mir-6873-3p,hsa-mir-6861-5p,hsa-mir-6848-3p,hsa-mir-6833-3p,hsa-mir-6817-3p,hsa-mir-6787-3p,hsa-mir-6783-3p,hsa-mir-6779-5p,hsa-mir-6775-3p,hsa-mir-6760-5p,hsa-mir-6742-3p,hsa-mir-6740-3p,hsa-mir-6736-3p,hsa-mir-664b-5p,hsa-mir-6500-5p,hsa-mir-629-3p,hsa-mir-6133,hsa-mir-6071,hsa-mir-5696,hsa-mir-5691,hsa-mir-554,hsa-mir-506-5p,hsa-mir-4775,hsa-mir-4770,hsa-mir-4768-5p,hsa-mir-4749-5p,hsa-mir-4743-3p,hsa-mir-4741,hsa-mir-4734,hsa-mir-4729,hsa-mir-4713-5p,hsa-mir-4711-3p,hsa-mir-4687-3p,hsa-mir-4675,hsa-mir-4660,hsa-mir-4659b-3p,hsa-mir-4659a-3p,hsa-mir-4650-5p,hsa-mir-4649-3p,hsa-mir-4633-3p,hsa-mir-4516,hsa-mir-4435,hsa-mir-4434,hsa-mir-4428,hsa-mir-4419a,hsa-mir-4303,hsa-mir-4283,hsa-mir-3977,hsa-mir-3936,hsa-mir-3934-5p,hsa-mir-3922-5p,hsa-mir-3689d,hsa-mir-3689c,hsa-mir-3689b-3p,hsa-mir-3689a-3p,hsa-mir-3685,hsa-mir-3675-5p,hsa-mir-3615,hsa-mir-3605-3p,hsa-mir-3192-5p,hsa-mir-3182,hsa-mir-3157-5p,hsa-mir-3156-5p,hsa-mir-3140-3p,hsa-mir-3139,hsa-mir-3125,hsa-mir-3124-3p,hsa-mir-3115,hsa-mir-30b-3p,hsa-mir-28-5p,hsa-mir-2467-5p,hsa-mir-211-3p,hsa-mir-2113,hsa-mir-185-3p,hsa-mir-148b-5p,hsa-mir-143-3p,hsa-mir-141-5p,hsa-mir-137,hsa-mir-1343-3p,hsa-mir-133b,hsa-mir-133a-3p,hsa-mir-1273h-5p,hsa-mir-1252-5p,hsa-mir-1237-3p,hsa-mir-122-5p |
| TPT1-AS1 | hsa-mir-99b-3p,hsa-mir-99a-3p,hsa-mir-9500,hsa-mir-943,hsa-mir-940,hsa-mir-939-5p,hsa-mir-939-3p,hsa-mir-890,hsa-mir-887-5p,hsa-mir-876-3p,hsa-mir-8085,hsa-mir-8068,hsa-mir-7977,hsa-mir-7856-5p,hsa-mir-7854-3p,hsa-mir-7843-5p,hsa-mir-770-5p,hsa-mir-7703,hsa-mir-769-3p,hsa-mir-767-3p,hsa-mir-765,hsa-mir-7-2-3p,hsa-mir-7160-5p,hsa-mir-7154-5p,hsa-mir-7154-3p,hsa-mir-7-1-3p,hsa-mir-7114-5p,hsa-mir-6893-5p,hsa-mir-6890-3p,hsa-mir-6888-3p,hsa-mir-6887-5p,hsa-mir-6887-3p,hsa-mir-6880-3p,hsa-mir-6879-5p,hsa-mir-6875-5p,hsa-mir-6873-3p,hsa-mir-6870-5p,hsa-mir-6867-5p,hsa-mir-6862-3p,hsa-mir-6860,hsa-mir-6855-5p,hsa-mir-6854-5p,hsa-mir-6852-5p,hsa-mir-6849-3p,hsa-mir-6847-5p,hsa-mir-6844,hsa-mir-6836-5p,hsa-mir-6833-3p,hsa-mir-6832-5p,hsa-mir-6828-3p,hsa-mir-6826-3p,hsa-mir-6825-5p,hsa-mir-6823-5p,hsa-mir-6823-3p,hsa-mir-6817-3p,hsa-mir-6811-3p,hsa-mir-6809-5p,hsa-mir-6808-5p,hsa-mir-6807-5p,hsa-mir-6796-3p,hsa-mir-6795-5p,hsa-mir-6793-5p,hsa-mir-6791-3p,hsa-mir-6790-3p,hsa-mir-6787-5p,hsa-mir-6780a-5p,hsa-mir-6779-5p,hsa-mir-6774-5p,hsa-mir-6769a-3p,hsa-mir-6765-3p,hsa-mir-6763-5p,hsa-mir-6759-5p,hsa-mir-6758-3p,hsa-mir-6754-3p,hsa-mir-6748-5p,hsa-mir-6745,hsa-mir-6740-3p,hsa-mir-6735-5p,hsa-mir-6730-5p,hsa-mir-6728-5p,hsa-mir-6727-3p,hsa-mir-6716-5p,hsa-mir-668-3p,hsa-mir-664b-3p,hsa-mir-661,hsa-mir-657,hsa-mir-656-5p,hsa-mir-655-5p,hsa-mir-654-3p,hsa-mir-6516-5p,hsa-mir-6515-3p,hsa-mir-6514-3p,hsa-mir-651-3p,hsa-mir-6513-5p,hsa-mir-6509-5p,hsa-mir-6506-5p,hsa-mir-6505-5p,hsa-mir-645,hsa-mir-635,hsa-mir-629-3p,hsa-mir-619-5p,hsa-mir-619-3p,hsa-mir-6165,hsa-mir-6134,hsa-mir-6132,hsa-mir-6127,hsa-mir-612,hsa-mir-6071,hsa-mir-605-3p,hsa-mir-590-3p,hsa-mir-585-5p,hsa-mir-582-3p,hsa-mir-579-3p,hsa-mir-576-5p,hsa-mir-5703,hsa-mir-5696,hsa-mir-5695,hsa-mir-5694,hsa-mir-5691,hsa-mir-5690,hsa-mir-5688,hsa-mir-5683,hsa-mir-5682,hsa-mir-5681a,hsa-mir-5590-3p,hsa-mir-5582-3p,hsa-mir-557,hsa-mir-551b-5p,hsa-mir-550b-3p,hsa-mir-548z,hsa-mir-548x-3p,hsa-mir-548p,hsa-mir-548j-3p,hsa-mir-548h-3p,hsa-mir-548g-3p,hsa-mir-548c-3p,hsa-mir-548av-3p,hsa-mir-548aq-3p,hsa-mir-548am-3p,hsa-mir-548aj-3p,hsa-mir-548ah-3p,hsa-mir-548ae-3p,hsa-mir-548ac,hsa-mir-543,hsa-mir-525-5p,hsa-mir-522-3p,hsa-mir-520a-5p,hsa-mir-519e-5p,hsa-mir-5197-3p,hsa-mir-518f-3p,hsa-mir-518a-3p,hsa-mir-515-5p,hsa-mir-513c-3p,hsa-mir-513a-3p,hsa-mir-510-5p,hsa-mir-5100,hsa-mir-5095,hsa-mir-5089-5p,hsa-mir-504-3p,hsa-mir-503-3p,hsa-mir-5004-5p,hsa-mir-5000-5p,hsa-mir-498,hsa-mir-495-3p,hsa-mir-494-3p,hsa-mir-4803,hsa-mir-4802-3p,hsa-mir-4775,hsa-mir-4768-5p,hsa-mir-4768-3p,hsa-mir-4762-5p,hsa-mir-4756-3p,hsa-mir-4755-3p,hsa-mir-4743-3p,hsa-mir-4736,hsa-mir-4731-5p,hsa-mir-4729,hsa-mir-4727-5p,hsa-mir-4722-5p,hsa-mir-4716-5p,hsa-mir-4713-5p,hsa-mir-4713-3p,hsa-mir-4712-5p,hsa-mir-4701-5p,hsa-mir-4699-5p,hsa-mir-4699-3p,hsa-mir-4694-5p,hsa-mir-4687-5p,hsa-mir-4686,hsa-mir-4684-5p,hsa-mir-4677-5p,hsa-mir-4667-5p,hsa-mir-4662b,hsa-mir-4662a-5p,hsa-mir-4660,hsa-mir-4653-3p,hsa-mir-4647,hsa-mir-4645-5p,hsa-mir-4632-3p,hsa-mir-4530,hsa-mir-4516,hsa-mir-450b-5p,hsa-mir-450b-3p,hsa-mir-4506,hsa-mir-449a,hsa-mir-4486,hsa-mir-4478,hsa-mir-4474-3p,hsa-mir-4470,hsa-mir-4468,hsa-mir-4443,hsa-mir-4439,hsa-mir-4436b-3p,hsa-mir-4434,hsa-mir-4430,hsa-mir-4429,hsa-mir-4426,hsa-mir-4425,hsa-mir-4420,hsa-mir-4322,hsa-mir-4320,hsa-mir-4318,hsa-mir-4314,hsa-mir-4312,hsa-mir-4309,hsa-mir-4306,hsa-mir-4294,hsa-mir-4282,hsa-mir-4272,hsa-mir-4265,hsa-mir-4264,hsa-mir-4258,hsa-mir-4257,hsa-mir-4254,hsa-mir-4252,hsa-mir-424-3p,hsa-mir-412-3p,hsa-mir-411-5p,hsa-mir-3942-3p,hsa-mir-3929,hsa-mir-3910,hsa-mir-374b-5p,hsa-mir-372-5p,hsa-mir-3714,hsa-mir-370-5p,hsa-mir-370-3p,hsa-mir-369-3p,hsa-mir-3692-5p,hsa-mir-3691-3p,hsa-mir-3690,hsa-mir-3689c,hsa-mir-3689b-3p,hsa-mir-3689a-3p,hsa-mir-3682-5p,hsa-mir-3679-3p,hsa-mir-3674,hsa-mir-3665,hsa-mir-3664-3p,hsa-mir-3662,hsa-mir-3652,hsa-mir-3650,hsa-mir-3613-3p,hsa-mir-3606-3p,hsa-mir-34a-5p,hsa-mir-345-5p,hsa-mir-335-3p,hsa-mir-330-3p,hsa-mir-320e,hsa-mir-320d,hsa-mir-320c,hsa-mir-320b,hsa-mir-320a,hsa-mir-3192-5p,hsa-mir-3187-5p,hsa-mir-3185,hsa-mir-3169,hsa-mir-3160-3p,hsa-mir-3156-5p,hsa-mir-3154,hsa-mir-3150a-3p,hsa-mir-3149,hsa-mir-3138,hsa-mir-3136-5p,hsa-mir-3135b,hsa-mir-3133,hsa-mir-3126-5p,hsa-mir-3122,hsa-mir-3121-3p,hsa-mir-3118,hsa-mir-3116,hsa-mir-30b-3p,hsa-mir-3074-5p,hsa-mir-2681-5p,hsa-mir-24-3p,hsa-mir-2278,hsa-mir-224-3p,hsa-mir-214-5p,hsa-mir-2116-3p,hsa-mir-2115-5p,hsa-mir-2110,hsa-mir-206,hsa-mir-205-3p,hsa-mir-204-3p,hsa-mir-197-3p,hsa-mir-193b-5p,hsa-mir-193a-3p,hsa-mir-186-3p,hsa-mir-185-5p,hsa-mir-183-3p,hsa-mir-181d-5p,hsa-mir-181c-5p,hsa-mir-181b-5p,hsa-mir-181b-2-3p,hsa-mir-181a-5p,hsa-mir-150-5p,hsa-mir-1-3p,hsa-mir-134-5p,hsa-mir-134-3p,hsa-mir-1343-5p,hsa-mir-1288-5p,hsa-mir-1277-5p,hsa-mir-1273h-5p,hsa-mir-1273g-3p,hsa-mir-1273f,hsa-mir-1255a,hsa-mir-1254,hsa-mir-1253,hsa-mir-1252-5p,hsa-mir-1249-5p,hsa-mir-1247-3p,hsa-mir-1243,hsa-mir-1229-5p,hsa-mir-1228-3p,hsa-mir-1226-5p,hsa-mir-1225-5p,hsa-mir-1203 |
