# Supplementary material for: Identification and validation of a five-lncRNA signature for predicting survival with targeted drug candidates in ovarian cancer
Source: Bioengineered. 2021 Jul 5;12(1):3263–74. doi: 10.1080/21655979.2021.1946632 (PMC8806566; doi:10.1080/21655979.2021.1946632)
Supplement: Supplemental Material [file KBIE_A_1946632_SM7043.zip › supplementary/Table S5.docx]

**Table S5. Target genes with | r | ≥ 0.3**

| **Gene** | **cor** |
| --- | --- |
| AARS2 | 0.316 |
| AASS | 0.3 |
| ABCA2 | 0.319 |
| ABCC10 | 0.3 |
| ABCC5 | 0.357 |
| ABCG8 | 0.309 |
| ABHD2 | -0.323 |
| ABHD5 | -0.33 |
| ABL1 | 0.308 |
| ACACB | 0.364 |
| ACAD11 | 0.307 |
| ACCS | 0.333 |
| ACIN1 | 0.368 |
| ACMSD | 0.379 |
| ACSBG2 | 0.356 |
| ACVR2B | 0.318 |
| ADAM33 | 0.418 |
| ADAMTS10 | 0.324 |
| ADAMTS13 | 0.378 |
| ADCK2 | -0.341 |
| ADCY6 | 0.306 |
| ADHFE1 | 0.353 |
| ADK | -0.342 |
| ADNP | 0.4 |
| ADRM1 | 0.304 |
| AGAP1 | 0.338 |
| AGAP4 | 0.414 |
| AGAP5 | 0.399 |
| AGAP6 | 0.39 |
| AGAP9 | 0.33 |
| AGBL2 | 0.305 |
| AGBL3 | 0.328 |
| AGER | 0.401 |
| AGO2 | 0.316 |
| AGPAT4 | 0.372 |
| AGRN | 0.302 |
| AGTR1 | -0.354 |
| AHDC1 | 0.33 |
| AK7 | 0.332 |
| AKAP11 | 0.47 |
| AKAP13 | 0.304 |
| ALG11 | 0.364 |
| ALS2 | 0.373 |
| AMBRA1 | 0.308 |
| AMMECR1L | 0.327 |
| AMT | 0.321 |
| ANAPC1 | 0.327 |
| ANGPT1 | -0.324 |
| ANKLE2 | 0.316 |
| ANKMY1 | 0.347 |
| ANKRD10 | 0.533 |
| ANKRD11 | 0.384 |
| ANKRD13A | -0.302 |
| ANKRD17 | 0.321 |
| ANKRD23 | 0.475 |
| ANKRD26 | 0.328 |
| ANKRD36 | 0.361 |
| ANKRD52 | 0.352 |
| ANKS1A | 0.35 |
| ANKS3 | 0.382 |
| ANKZF1 | 0.334 |
| ANLN | 0.368 |
| ANO7 | 0.301 |
| ANP32B | 0.41 |
| ANP32E | 0.306 |
| ANXA7 | -0.311 |
| AOC3 | -0.323 |
| AP1G1 | 0.314 |
| AP2S1 | 0.339 |
| APBB3 | 0.37 |
| APEX2 | -0.314 |
| APOO | 0.306 |
| ARAP1 | 0.332 |
| ARAP3 | 0.308 |
| ARFGAP1 | 0.323 |
| ARFGEF2 | 0.358 |
| ARGLU1 | 0.55 |
| ARHGAP11A | 0.35 |
| ARHGAP39 | 0.31 |
| ARHGEF1 | 0.341 |
| ARHGEF11 | 0.327 |
| ARHGEF17 | 0.36 |
| ARHGEF18 | -0.302 |
| ARHGEF39 | 0.358 |
| ARHGEF7 | 0.351 |
| ARID1A | 0.377 |
| ARL13A | 0.305 |
| ARL17A | 0.368 |
| ARL6IP1 | 0.351 |
| ARMCX4 | 0.429 |
| ARNT | 0.33 |
| ARPC4 | -0.358 |
| ARPC4-TTLL3 | 0.351 |
| ARPC5L | -0.309 |
| ARSD | 0.335 |
| ARSI | -0.324 |
| ARVCF | 0.327 |
| ASAP2 | 0.349 |
| ASB16 | 0.405 |
| ASB3 | 0.317 |
| ASF1B | 0.378 |
| ASH1L | 0.414 |
| ASPDH | 0.313 |
| ASPM | 0.414 |
| ASPN | -0.336 |
| ASPRV1 | 0.425 |
| ASXL1 | 0.405 |
| ASXL2 | 0.363 |
| ATAD2B | 0.345 |
| ATAD5 | 0.35 |
| ATAT1 | 0.398 |
| ATF7 | 0.327 |
| ATG16L2 | 0.346 |
| ATM | 0.33 |
| ATMIN | 0.33 |
| ATP2A1 | 0.364 |
| ATP6AP1L | 0.336 |
| ATP6AP2 | -0.347 |
| ATP6V0A1 | 0.332 |
| ATP6V0B | -0.354 |
| ATP6V0E1 | -0.381 |
| ATP6V1F | -0.326 |
| ATP7B | 0.305 |
| ATP8B2 | 0.301 |
| ATRN | 0.346 |
| ATXN1L | 0.346 |
| ATXN2 | 0.317 |
| ATXN2L | 0.369 |
| ATXN7L2 | 0.348 |
| ATXN7L3 | 0.324 |
| AUNIP | 0.332 |
| AURKA | 0.306 |
| B2M | -0.317 |
| B3GALNT2 | 0.361 |
| BANF1 | 0.3 |
| BARD1 | 0.35 |
| BAZ1A | 0.34 |
| BAZ2A | 0.329 |
| BBIP1 | 0.302 |
| BBS1 | 0.46 |
| BCAP31 | -0.315 |
| BCDIN3D | 0.319 |
| BCL9 | 0.326 |
| BCL9L | -0.319 |
| BCO2 | 0.334 |
| BCOR | 0.327 |
| BGLAP | 0.501 |
| BGN | -0.336 |
| BICD2 | 0.306 |
| BIRC5 | 0.402 |
| BIRC6 | 0.326 |
| BLOC1S1 | 0.329 |
| BMPR1B | 0.608 |
| BOD1L1 | 0.337 |
| BOLA3 | -0.359 |
| BPTF | 0.369 |
| BRD3 | 0.322 |
| BRIX1 | 0.377 |
| BRK1 | -0.313 |
| BRPF3 | 0.32 |
| BSPRY | -0.332 |
| BTAF1 | 0.44 |
| BTBD18 | 0.366 |
| BTBD3 | 0.309 |
| BTBD9 | 0.338 |
| BUB1 | 0.376 |
| BUB3 | 0.344 |
| C16orf46 | 0.367 |
| C16orf58 | -0.378 |
| C19orf44 | 0.322 |
| C19orf73 | 0.313 |
| C1orf122 | -0.356 |
| C1orf158 | 0.384 |
| C1orf189 | 0.304 |
| C1orf210 | -0.305 |
| C1orf43 | -0.308 |
| C20orf194 | 0.363 |
| C20orf96 | 0.342 |
| C5orf49 | 0.391 |
| C6orf118 | 0.376 |
| C7orf61 | 0.309 |
| CAB39L | 0.377 |
| CACNB1 | 0.368 |
| CACNB2 | 0.455 |
| CAD | 0.309 |
| CAMSAP1 | 0.361 |
| CAMTA1 | 0.32 |
| CAMTA2 | -0.312 |
| CAP1 | -0.324 |
| CAPN10 | 0.347 |
| CAPN3 | 0.386 |
| CAPRIN2 | 0.38 |
| CARD10 | 0.3 |
| CARD8 | 0.316 |
| CARF | 0.352 |
| CASKIN1 | 0.302 |
| CATSPER2 | 0.403 |
| CATSPER4 | 0.34 |
| CBFA2T2 | 0.407 |
| CBFB | 0.304 |
| CBR1 | -0.383 |
| CC2D2B | 0.398 |
| CCDC107 | -0.314 |
| CCDC113 | 0.312 |
| CCDC114 | 0.445 |
| CCDC122 | 0.394 |
| CCDC14 | 0.398 |
| CCDC173 | 0.347 |
| CCDC18 | 0.348 |
| CCDC30 | 0.312 |
| CCDC33 | 0.305 |
| CCDC34 | 0.331 |
| CCDC40 | 0.47 |
| CCDC58 | 0.327 |
| CCDC62 | 0.307 |
| CCDC70 | 0.336 |
| CCDC80 | -0.348 |
| CCDC84 | 0.311 |
| CCDC93 | 0.396 |
| CCDC96 | 0.322 |
| CCNB1 | 0.373 |
| CCNL1 | 0.332 |
| CCNL2 | 0.401 |
| CCNT2 | 0.375 |
| CD248 | -0.31 |
| CD36 | -0.324 |
| CD3EAP | 0.337 |
| CD63 | -0.31 |
| CDADC1 | 0.403 |
| CDC16 | 0.35 |
| CDC25A | 0.321 |
| CDC25C | 0.334 |
| CDC45 | 0.367 |
| CDC7 | 0.315 |
| CDCA3 | 0.384 |
| CDCA5 | 0.305 |
| CDCA7 | 0.35 |
| CDCA8 | 0.31 |
| CDK1 | 0.449 |
| CDK10 | 0.302 |
| CDK12 | 0.348 |
| CDK13 | 0.305 |
| CDK19 | 0.323 |
| CDK20 | 0.303 |
| CDK3 | 0.398 |
| CDKN3 | 0.479 |
| CDRT4 | 0.495 |
| CDT1 | 0.422 |
| CEACAM19 | 0.31 |
| CELF1 | 0.344 |
| CELSR1 | 0.32 |
| CENPA | 0.419 |
| CENPF | 0.371 |
| CENPJ | 0.384 |
| CENPK | 0.311 |
| CENPM | 0.332 |
| CENPN | 0.559 |
| CEP250 | 0.365 |
| CEP55 | 0.446 |
| CEP95 | 0.3 |
| CEP97 | 0.303 |
| CFDP1 | 0.302 |
| CFL1 | 0.31 |
| CHAC2 | 0.365 |
| CHAF1A | 0.315 |
| CHAF1B | 0.304 |
| CHCHD1 | -0.341 |
| CHD1 | 0.307 |
| CHD2 | 0.388 |
| CHD4 | 0.3 |
| CHD6 | 0.415 |
| CHD7 | 0.302 |
| CHD8 | 0.3 |
| CHEK1 | 0.313 |
| CHMP4A | 0.3 |
| CIC | 0.364 |
| CIRBP | 0.335 |
| CKAP2L | 0.348 |
| CKLF | -0.322 |
| CKS2 | 0.47 |
| CLASP1 | 0.382 |
| CLCN2 | 0.304 |
| CLCN6 | 0.444 |
| CLCN7 | 0.321 |
| CLDN20 | 0.302 |
| CLEC16A | 0.307 |
| CLEC1A | -0.303 |
| CLHC1 | 0.45 |
| CLK1 | 0.419 |
| CLK2 | 0.313 |
| CLK4 | 0.368 |
| CLMP | -0.359 |
| CLPB | -0.333 |
| CLSPN | 0.346 |
| CMTM1 | 0.34 |
| CMTM4 | 0.302 |
| CNNM3 | 0.34 |
| CNNM4 | 0.301 |
| CNOT1 | 0.325 |
| CNST | 0.307 |
| CNTNAP1 | 0.346 |
| CNTRL | 0.3 |
| CNTROB | 0.3 |
| COA4 | -0.323 |
| COG3 | 0.512 |
| COG7 | 0.306 |
| COL5A2 | -0.301 |
| COL7A1 | 0.345 |
| COLGALT1 | -0.3 |
| COLQ | 0.486 |
| COMTD1 | -0.309 |
| COPZ2 | -0.332 |
| COQ2 | -0.327 |
| CORT | 0.381 |
| COX14 | -0.305 |
| COX17 | -0.348 |
| COX7A2 | -0.301 |
| COX8A | -0.301 |
| CPSF6 | 0.317 |
| CPSF7 | 0.355 |
| CPT2 | -0.319 |
| CREB1 | 0.358 |
| CREBBP | 0.375 |
| CRISPLD2 | -0.3 |
| CRKL | 0.314 |
| CROCC | 0.395 |
| CRTC1 | 0.319 |
| CRY2 | -0.368 |
| CSAD | 0.335 |
| CSNK1A1 | -0.333 |
| CSNK2A1 | 0.334 |
| CTC1 | 0.323 |
| CTCF | 0.344 |
| CTDSP2 | -0.306 |
| CTNND1 | 0.315 |
| CTSC | -0.346 |
| CTSL | -0.342 |
| CUBN | 0.407 |
| CXCL10 | -0.311 |
| CXCL12 | -0.321 |
| CYB5B | 0.332 |
| CYB5D1 | -0.381 |
| CYP2C8 | 0.36 |
| CYP2E1 | 0.313 |
| CYP3A43 | 0.312 |
| D2HGDH | 0.336 |
| DAB2IP | 0.337 |
| DACT1 | -0.311 |
| DACT3 | -0.364 |
| DBF4 | 0.399 |
| DBF4B | 0.351 |
| DBI | -0.337 |
| DCAF16 | 0.348 |
| DCAF5 | 0.312 |
| DCN | -0.322 |
| DCTN4 | -0.318 |
| DCUN1D2 | 0.432 |
| DDX17 | 0.432 |
| DDX19B | 0.314 |
| DDX39A | 0.321 |
| DDX39B | 0.419 |
| DDX42 | 0.364 |
| DENND4B | 0.379 |
| DEPDC1 | 0.368 |
| DEPDC5 | 0.358 |
| DGCR8 | 0.433 |
| DGKD | 0.353 |
| DGKH | 0.371 |
| DHRS12 | 0.403 |
| DHX38 | 0.32 |
| DIAPH3 | 0.308 |
| DIDO1 | 0.408 |
| DIS3 | 0.352 |
| DLEC1 | 0.326 |
| DMC1 | 0.403 |
| DMTF1 | 0.382 |
| DNAH17 | 0.333 |
| DNAH9 | 0.303 |
| DNAJC11 | 0.328 |
| DNAJC16 | 0.324 |
| DNAJC27 | 0.371 |
| DNMT3A | 0.329 |
| DONSON | 0.338 |
| DOT1L | 0.363 |
| DPH1 | -0.38 |
| DPP10 | 0.307 |
| DRD2 | 0.311 |
| DSCC1 | 0.324 |
| DUOX1 | 0.321 |
| DUSP23 | -0.305 |
| DVL3 | 0.32 |
| DYDC2 | 0.307 |
| DYNC1LI2 | 0.373 |
| DYNLL1 | 0.327 |
| DZANK1 | 0.356 |
| DZIP3 | 0.318 |
| EBLN2 | 0.367 |
| EBNA1BP2 | -0.339 |
| ECE1 | -0.317 |
| ECM2 | -0.301 |
| ECT2 | 0.348 |
| EDC4 | 0.367 |
| EDF1 | -0.306 |
| EDNRA | -0.3 |
| EEF1A1 | -0.327 |
| EEF2 | -0.378 |
| EFCAB13 | 0.41 |
| EFEMP1 | -0.333 |
| EFHC1 | 0.342 |
| EFHC2 | 0.348 |
| EGFL8 | 0.522 |
| EHMT1 | 0.32 |
| EIF2B3 | -0.339 |
| EIF3L | -0.435 |
| EIF4A1 | 0.307 |
| EIF4B | -0.301 |
| EIF4EBP1 | 0.311 |
| EIF4ENIF1 | 0.306 |
| EIF4G1 | 0.334 |
| EIF4G3 | 0.327 |
| EIF5B | 0.345 |
| ELF2 | 0.328 |
| ELMO2 | 0.306 |
| ELMOD3 | 0.306 |
| ELMSAN1 | 0.31 |
| ELOVL1 | -0.343 |
| EMC3 | -0.327 |
| EMC7 | -0.317 |
| EMC8 | 0.364 |
| EMX2 | 0.304 |
| ENDOV | 0.314 |
| ENO4 | 0.3 |
| ENOSF1 | 0.302 |
| ENOX1 | -0.33 |
| ENTPD4 | 0.321 |
| EP300 | 0.4 |
| EP400 | 0.417 |
| EPB41 | 0.325 |
| EPC1 | 0.305 |
| EPC2 | 0.326 |
| EPG5 | 0.316 |
| EPN2 | -0.382 |
| EPOR | 0.448 |
| EPPK1 | 0.339 |
| ERCC5 | 0.345 |
| ERCC6L | 0.388 |
| ERCC6L2 | 0.33 |
| ERGIC1 | -0.308 |
| ERP44 | -0.363 |
| ERVW-1 | 0.389 |
| ESCO2 | 0.342 |
| ESD | 0.352 |
| EVC | -0.339 |
| EXO1 | 0.322 |
| EXOC7 | -0.367 |
| EXOSC6 | 0.38 |
| EXOSC8 | 0.325 |
| EXPH5 | 0.306 |
| EZH1 | 0.404 |
| EZH2 | 0.371 |
| F13A1 | -0.35 |
| FAHD2A | 0.307 |
| FAM111A | 0.309 |
| FAM111B | 0.326 |
| FAM120B | 0.324 |
| FAM124A | 0.414 |
| FAM136A | 0.303 |
| FAM160A1 | 0.346 |
| FAM160A2 | 0.331 |
| FAM160B2 | 0.341 |
| FAM166B | 0.3 |
| FAM168B | 0.372 |
| FAM184B | 0.391 |
| FAM186A | 0.342 |
| FAM186B | 0.398 |
| FAM193A | 0.317 |
| FAM193B | 0.375 |
| FAM19A2 | 0.314 |
| FAM219B | -0.323 |
| FAM227A | 0.46 |
| FAM228B | 0.384 |
| FAM72A | 0.389 |
| FAM72B | 0.351 |
| FAM72C | 0.41 |
| FAM72D | 0.44 |
| FAM81B | 0.407 |
| FAM83G | -0.319 |
| FAM86B1 | 0.356 |
| FANCA | 0.323 |
| FARP1 | 0.4 |
| FARP2 | 0.327 |
| FBN1 | -0.328 |
| FBN3 | 0.468 |
| FBP1 | -0.327 |
| FBRS | 0.405 |
| FBRSL1 | 0.329 |
| FBXL18 | 0.317 |
| FBXL19 | 0.326 |
| FBXL2 | 0.378 |
| FBXL20 | 0.3 |
| FBXL3 | 0.37 |
| FBXO11 | 0.372 |
| FBXO24 | 0.304 |
| FBXW11 | -0.355 |
| FBXW8 | 0.335 |
| FEN1 | 0.305 |
| FGF7 | -0.341 |
| FGFR1OP | 0.348 |
| FHOD1 | 0.309 |
| FLCN | 0.35 |
| FLYWCH1 | 0.31 |
| FMNL2 | 0.358 |
| FNBP4 | 0.365 |
| FNDC3A | 0.43 |
| FOXD4L5 | 0.302 |
| FOXJ1 | 0.676 |
| FOXK1 | 0.372 |
| FPGT | -0.317 |
| FPGT-TNNI3K | 0.336 |
| FTL | -0.327 |
| FUNDC1 | 0.374 |
| FXYD1 | 0.327 |
| FZD3 | 0.335 |
| GALNT6 | 0.315 |
| GARNL3 | 0.316 |
| GATAD2B | 0.338 |
| GCC2 | 0.308 |
| GGA1 | 0.328 |
| GIGYF1 | 0.392 |
| GIGYF2 | 0.368 |
| GINS1 | 0.315 |
| GINS2 | 0.528 |
| GIT2 | -0.31 |
| GJC2 | 0.342 |
| GLG1 | 0.304 |
| GLIPR1 | -0.309 |
| GLRX2 | -0.335 |
| GMEB2 | 0.308 |
| GNG2 | -0.316 |
| GNG5 | -0.346 |
| GNPTAB | -0.303 |
| GOLGA3 | 0.378 |
| GOLGA6L9 | 0.301 |
| GOLGA8A | 0.372 |
| GOLGA8B | 0.394 |
| GOLGA8R | 0.301 |
| GON4L | 0.389 |
| GOPC | 0.313 |
| GOSR1 | 0.378 |
| GPALPP1 | 0.567 |
| GPATCH2 | 0.304 |
| GPATCH2L | 0.309 |
| GPATCH8 | 0.416 |
| GPCPD1 | 0.307 |
| GPR179 | 0.315 |
| GPR19 | 0.335 |
| GPR35 | 0.353 |
| GPRASP1 | 0.321 |
| GRM6 | 0.383 |
| GSKIP | -0.336 |
| GTF2F2 | 0.485 |
| GTF2I | 0.343 |
| GTF2IRD2 | 0.379 |
| GTF2IRD2B | 0.311 |
| GTF3C1 | 0.383 |
| GTSE1 | 0.305 |
| GUCA1B | 0.374 |
| GYG1 | -0.356 |
| GZF1 | 0.343 |
| H1FNT | 0.363 |
| H2AFJ | -0.361 |
| H2AFZ | 0.337 |
| HAND2 | -0.314 |
| HAUS8 | 0.37 |
| HBP1 | -0.318 |
| HCCS | -0.407 |
| HCFC1 | 0.385 |
| HCN3 | 0.304 |
| HDAC4 | 0.331 |
| HDAC5 | -0.324 |
| HDAC6 | 0.34 |
| HEATR5B | 0.337 |
| HECTD4 | 0.419 |
| HELLS | 0.322 |
| HELZ | 0.362 |
| HEMK1 | 0.337 |
| HERC1 | 0.33 |
| HERC2 | 0.31 |
| HIC1 | -0.308 |
| HIC2 | 0.393 |
| HIF1AN | 0.322 |
| HIF3A | 0.361 |
| HIGD2A | -0.329 |
| HINT1 | -0.335 |
| HIP1R | 0.307 |
| HIST1H2AJ | 0.331 |
| HIST1H2BJ | 0.3 |
| HIST1H2BO | 0.325 |
| HIST1H3B | 0.316 |
| HIST1H3C | 0.308 |
| HIST1H3F | 0.335 |
| HIST1H3G | 0.314 |
| HIST1H4C | 0.347 |
| HIST3H3 | 0.3 |
| HIVEP2 | 0.317 |
| HJURP | 0.355 |
| HMBOX1 | 0.427 |
| HMG20A | 0.34 |
| HMGB2 | 0.452 |
| HMGB3 | 0.385 |
| HMGN5 | 0.302 |
| HMMR | 0.318 |
| HNRNPA1L2 | 0.6 |
| HNRNPDL | 0.357 |
| HNRNPH1 | 0.379 |
| HNRNPU | 0.316 |
| HNRNPUL2 | 0.303 |
| HP1BP3 | 0.32 |
| HPRT1 | -0.38 |
| HPS4 | 0.377 |
| HSBP1 | 0.419 |
| HSPG2 | -0.348 |
| HYDIN | 0.372 |
| HYPK | 0.315 |
| IBA57 | 0.303 |
| ICA1L | 0.325 |
| ID3 | -0.365 |
| IDH2 | -0.31 |
| IDH3A | -0.307 |
| IFI35 | -0.304 |
| IFI6 | 0.3 |
| IFT140 | 0.34 |
| IFT172 | 0.329 |
| IFT80 | 0.345 |
| IFT81 | 0.311 |
| IGF2R | 0.3 |
| IGHMBP2 | 0.347 |
| IL11RA | 0.35 |
| IL5 | 0.347 |
| IL5RA | 0.312 |
| ILDR1 | 0.325 |
| ILF3 | 0.306 |
| ILK | -0.337 |
| ING1 | 0.316 |
| ING5 | 0.368 |
| INO80D | 0.387 |
| INPP4A | 0.326 |
| INTS2 | 0.31 |
| INTS3 | 0.408 |
| INTS6 | 0.396 |
| INTU | 0.37 |
| IP6K2 | 0.321 |
| IQGAP3 | 0.354 |
| IQSEC1 | 0.332 |
| IRAK1 | -0.3 |
| IREB2 | 0.311 |
| IRGQ | 0.308 |
| IRS2 | 0.337 |
| ISY1 | 0.333 |
| JARID2 | 0.327 |
| JMJD7 | 0.363 |
| JMJD7-PLA2G4B | 0.409 |
| JTB | -0.342 |
| KANSL1 | 0.436 |
| KANSL1L | 0.336 |
| KANSL3 | 0.447 |
| KARS | 0.339 |
| KAT2A | 0.378 |
| KAT6A | 0.362 |
| KAT6B | 0.329 |
| KAT7 | 0.352 |
| KBTBD4 | -0.369 |
| KBTBD6 | 0.417 |
| KBTBD7 | 0.407 |
| KCNAB3 | 0.335 |
| KCNE4 | -0.36 |
| KCNIP2 | 0.355 |
| KCTD11 | -0.312 |
| KCTD7 | 0.361 |
| KDM2A | 0.351 |
| KDM2B | 0.358 |
| KDM3A | 0.315 |
| KDM3B | 0.353 |
| KDM6B | 0.352 |
| KIAA0100 | -0.348 |
| KIAA0232 | 0.327 |
| KIAA0355 | 0.32 |
| KIAA0556 | 0.452 |
| KIAA0753 | 0.358 |
| KIAA0895L | 0.397 |
| KIAA1109 | 0.402 |
| KIAA1217 | 0.31 |
| KIAA1614 | 0.319 |
| KIDINS220 | 0.332 |
| KIF11 | 0.427 |
| KIF14 | 0.323 |
| KIF18A | 0.38 |
| KIF1B | 0.322 |
| KIF20B | 0.403 |
| KIF23 | 0.458 |
| KIF27 | 0.325 |
| KIF2C | 0.325 |
| KIF4A | 0.359 |
| KLF12 | 0.476 |
| KLHL11 | 0.387 |
| KLHL31 | 0.349 |
| KMT2A | 0.321 |
| KMT2B | 0.356 |
| KMT2C | 0.312 |
| KMT2D | 0.404 |
| KMT2E | 0.355 |
| KNSTRN | 0.314 |
| KPNA2 | 0.341 |
| KPNA5 | 0.352 |
| KRBA2 | 0.333 |
| KRTAP5-7 | 0.344 |
| KRTAP5-8 | 0.314 |
| KRTAP5-9 | 0.314 |
| KSR1 | 0.385 |
| L3MBTL1 | 0.479 |
| LACTB | -0.359 |
| LAMA4 | -0.323 |
| LAMA5 | 0.355 |
| LAMTOR1 | -0.343 |
| LAMTOR5 | -0.355 |
| LAP3 | -0.317 |
| LARP7 | 0.306 |
| LATS2 | 0.331 |
| LCAT | 0.367 |
| LCN12 | 0.355 |
| LDLRAD1 | 0.313 |
| LDLRAD2 | 0.353 |
| LEKR1 | 0.328 |
| LENEP | 0.303 |
| LENG8 | 0.389 |
| LGI4 | 0.31 |
| LHX4 | 0.368 |
| LIPI | 0.32 |
| LMBR1L | 0.356 |
| LMLN | 0.348 |
| LMO7 | 0.311 |
| LMTK2 | 0.39 |
| LOX | -0.355 |
| LPCAT3 | -0.366 |
| LRBA | 0.317 |
| LRCH1 | 0.303 |
| LRCH3 | 0.349 |
| LRCH4 | 0.382 |
| LRIG2 | 0.306 |
| LRP1 | -0.32 |
| LRRC39 | 0.326 |
| LRRC71 | 0.31 |
| LRRIQ1 | 0.396 |
| LSM10 | -0.359 |
| LSM11 | -0.351 |
| LSM3 | 0.389 |
| LSM4 | 0.337 |
| LSM5 | 0.331 |
| LSMEM1 | 0.344 |
| LSMEM2 | 0.329 |
| LUC7L | 0.5 |
| LUC7L3 | 0.336 |
| LY6G5B | 0.442 |
| LYN | -0.317 |
| LYPD6B | 0.356 |
| M6PR | -0.303 |
| MAATS1 | 0.37 |
| MAD2L1 | 0.32 |
| MAGOH | 0.406 |
| MAGOHB | 0.356 |
| MAK | 0.399 |
| MAML3 | 0.347 |
| MAN2A2 | 0.317 |
| MAPK8IP3 | 0.476 |
| MAPKBP1 | -0.322 |
| MARVELD3 | 0.348 |
| MASP2 | 0.436 |
| MAST3 | 0.336 |
| MASTL | 0.389 |
| MAT2A | 0.35 |
| MATR3 | 0.346 |
| MAU2 | 0.354 |
| MAVS | 0.357 |
| MBD2 | 0.358 |
| MBD5 | 0.377 |
| MBNL2 | 0.387 |
| MBTD1 | 0.35 |
| MCM10 | 0.318 |
| MCM3AP | 0.305 |
| MDC1 | 0.304 |
| MDM4 | 0.429 |
| MDN1 | 0.366 |
| MECOM | 0.307 |
| MED1 | 0.358 |
| MED12 | 0.311 |
| MED13 | 0.367 |
| MED13L | 0.333 |
| MED4 | 0.451 |
| MEF2A | 0.315 |
| MEF2D | -0.34 |
| MEGF8 | 0.336 |
| MEIS3 | -0.32 |
| MELK | 0.427 |
| METAP1D | 0.321 |
| METTL14 | 0.314 |
| MFAP3 | -0.392 |
| MGA | 0.407 |
| MGAM | 0.307 |
| MGAT5 | 0.304 |
| MICAL3 | 0.351 |
| MINK1 | -0.307 |
| MIS18A | 0.454 |
| MKS1 | 0.304 |
| MLLT6 | 0.357 |
| MLXIP | 0.302 |
| MND1 | 0.535 |
| MNT | 0.36 |
| MORC2 | 0.316 |
| MOXD1 | -0.361 |
| MPC2 | -0.3 |
| MPHOSPH6 | 0.495 |
| MPHOSPH8 | 0.371 |
| MPP3 | 0.31 |
| MPRIP | 0.35 |
| MRPL24 | -0.304 |
| MRPL37 | -0.39 |
| MRPL51 | -0.33 |
| MRPS15 | -0.385 |
| MRPS16 | -0.306 |
| MSANTD2 | 0.444 |
| MSC | -0.342 |
| MSH5 | 0.414 |
| MSL1 | 0.302 |
| MSL2 | 0.305 |
| MSMO1 | -0.324 |
| MSRB3 | -0.309 |
| MSS51 | 0.386 |
| MTHFR | 0.303 |
| MTMR4 | 0.33 |
| MTOR | 0.329 |
| MTR | 0.31 |
| MTRF1 | 0.45 |
| MTRNR2L4 | 0.307 |
| MTX3 | 0.353 |
| MYBL2 | 0.403 |
| MYCBP | -0.307 |
| MYCBP2 | 0.36 |
| MYEF2 | 0.355 |
| MYH7B | 0.345 |
| MYO15A | 0.334 |
| MYO9B | -0.315 |
| N4BP2L1 | 0.32 |
| N4BP2L2 | 0.481 |
| NAA16 | 0.601 |
| NAA25 | 0.338 |
| NAA40 | 0.424 |
| NAALADL1 | 0.353 |
| NAPB | 0.407 |
| NASP | 0.331 |
| NBEAL2 | 0.327 |
| NBPF1 | 0.347 |
| NBPF11 | 0.432 |
| NBPF12 | 0.421 |
| NBPF9 | 0.311 |
| NBR1 | -0.36 |
| NCAPG | 0.356 |
| NCAPH | 0.352 |
| NCOA5 | 0.343 |
| NCOA6 | 0.368 |
| NCOR1 | 0.331 |
| NCOR2 | 0.383 |
| NDST1 | -0.334 |
| NDUFA12 | -0.354 |
| NDUFA2 | -0.342 |
| NDUFA7 | -0.303 |
| NDUFAF3 | -0.313 |
| NDUFB4 | -0.328 |
| NDUFB6 | -0.32 |
| NDUFC2 | -0.3 |
| NDUFS5 | -0.32 |
| NEIL3 | 0.395 |
| NEK11 | 0.328 |
| NEK3 | 0.469 |
| NEK5 | 0.473 |
| NEK9 | 0.322 |
| NEURL4 | 0.367 |
| NFAT5 | 0.438 |
| NFATC3 | 0.357 |
| NFRKB | 0.323 |
| NFYA | 0.32 |
| NHLRC3 | 0.31 |
| NHP2 | -0.304 |
| NIPBL | 0.317 |
| NISCH | 0.325 |
| NKTR | 0.472 |
| NLGN2 | 0.321 |
| NME9 | 0.395 |
| NMI | -0.315 |
| NOL9 | 0.328 |
| NOP58 | 0.309 |
| NOS1AP | 0.352 |
| NPAS3 | 0.344 |
| NPHP1 | 0.309 |
| NPHP3 | 0.435 |
| NPHP4 | 0.399 |
| NQO1 | -0.347 |
| NR2C2 | 0.381 |
| NRF1 | 0.366 |
| NRIP2 | 0.353 |
| NSD1 | 0.328 |
| NSDHL | -0.371 |
| NTN5 | 0.382 |
| NUAK2 | 0.319 |
| NUDT13 | 0.304 |
| NUDT15 | 0.32 |
| NUDT8 | -0.344 |
| NUF2 | 0.455 |
| NUFIP1 | 0.545 |
| NUMA1 | 0.324 |
| NUSAP1 | 0.403 |
| NUTF2 | 0.451 |
| NXF1 | 0.389 |
| OAT | -0.332 |
| OAZ1 | -0.32 |
| OBSCN | 0.407 |
| OGFOD2 | 0.342 |
| OGT | 0.301 |
| OMD | -0.322 |
| ORC6 | 0.417 |
| ORMDL2 | -0.34 |
| OSBPL2 | 0.319 |
| OST4 | -0.341 |
| OSTF1 | -0.355 |
| OTUD3 | 0.323 |
| OTUD4 | 0.38 |
| PA2G4 | 0.391 |
| PABPC1L | 0.416 |
| PACRG | 0.327 |
| PAN2 | 0.369 |
| PAN3 | 0.457 |
| PAPSS2 | -0.307 |
| PAQR6 | 0.319 |
| PASK | 0.352 |
| PBK | 0.415 |
| PBRM1 | 0.3 |
| PCBD1 | -0.301 |
| PCDHGC3 | -0.341 |
| PCED1A | 0.345 |
| PCIF1 | 0.311 |
| PCMTD2 | 0.31 |
| PCNA | 0.337 |
| PDC | 0.309 |
| PDK2 | -0.339 |
| PDLIM3 | -0.305 |
| PDPK1 | 0.356 |
| PDZD11 | -0.347 |
| PDZD9 | 0.304 |
| PEAK1 | 0.302 |
| PER2 | -0.3 |
| PER3 | 0.318 |
| PEX12 | -0.302 |
| PFDN6 | 0.305 |
| PFKFB2 | 0.307 |
| PFN1 | -0.375 |
| PGAM1 | -0.302 |
| PGK1 | -0.371 |
| PGS1 | 0.329 |
| PHC3 | 0.346 |
| PHF1 | 0.305 |
| PHF10 | 0.341 |
| PHF12 | 0.355 |
| PHF2 | 0.317 |
| PHF21A | 0.329 |
| PHGDH | 0.306 |
| PHKG1 | 0.404 |
| PHRF1 | 0.303 |
| PHYKPL | 0.335 |
| PI4KA | 0.301 |
| PIBF1 | 0.351 |
| PIFO | 0.431 |
| PIGS | -0.311 |
| PIK3R2 | 0.321 |
| PIKFYVE | 0.386 |
| PILRB | 0.337 |
| PIN4 | 0.385 |
| PIP4K2B | 0.342 |
| PIP5K1A | 0.307 |
| PIR | -0.343 |
| PIWIL2 | 0.43 |
| PKD1 | 0.376 |
| PKIA | 0.3 |
| PKMYT1 | 0.327 |
| PLA2G15 | -0.301 |
| PLA2G4B | 0.355 |
| PLA2G6 | 0.397 |
| PLAGL2 | 0.316 |
| PLCG1 | 0.396 |
| PLEKHA7 | 0.334 |
| PLEKHG2 | 0.316 |
| PLEKHG3 | 0.344 |
| PLEKHG4 | 0.345 |
| PLEKHG5 | 0.352 |
| PLEKHH1 | 0.305 |
| PLEKHM3 | 0.407 |
| PLGLB1 | 0.377 |
| PLGRKT | 0.336 |
| PLN | -0.326 |
| PLXNB1 | 0.356 |
| PMFBP1 | 0.351 |
| PMP22 | -0.315 |
| PMS2 | 0.3 |
| PNISR | 0.487 |
| PNMA3 | 0.311 |
| PNPLA7 | 0.359 |
| POGZ | 0.436 |
| POLDIP3 | -0.313 |
| POLE | 0.318 |
| POLE2 | 0.342 |
| POLM | 0.305 |
| POLQ | 0.308 |
| POLR1A | 0.36 |
| POLR2A | 0.316 |
| POLR2J | -0.3 |
| POLR2L | -0.313 |
| POLR3E | 0.334 |
| POM121 | 0.36 |
| POM121C | 0.351 |
| POMK | 0.363 |
| POP7 | -0.32 |
| POPDC2 | 0.326 |
| POU2F1 | 0.334 |
| PPA1 | -0.325 |
| PPCS | -0.334 |
| PPFIA4 | 0.341 |
| PPIA | -0.308 |
| PPP5D1 | 0.424 |
| PRCD | 0.375 |
| PRDM10 | 0.389 |
| PRDM11 | 0.307 |
| PRDM15 | 0.305 |
| PRDM4 | 0.309 |
| PRDX1 | -0.344 |
| PRELID1 | -0.324 |
| PRICKLE4 | 0.318 |
| PROX2 | 0.309 |
| PRPF40B | 0.366 |
| PRPF4B | 0.331 |
| PRR12 | 0.382 |
| PRR14L | 0.38 |
| PRRC2A | 0.354 |
| PRRC2B | 0.379 |
| PRRC2C | 0.322 |
| PRRT2 | 0.374 |
| PRRX1 | -0.365 |
| PRSS37 | 0.345 |
| PRSS42 | 0.304 |
| PRSS53 | 0.354 |
| PSMA2 | 0.505 |
| PSMA3 | 0.31 |
| PSMA5 | -0.403 |
| PSMA6 | -0.323 |
| PSMC3IP | 0.305 |
| PSMD14 | 0.305 |
| PSME1 | -0.314 |
| PSMG1 | 0.395 |
| PSMG2 | 0.312 |
| PTCD2 | 0.365 |
| PTCH1 | 0.409 |
| PTGFR | -0.328 |
| PTGIS | -0.304 |
| PTMA | 0.307 |
| PTPN14 | 0.304 |
| PTPRU | -0.373 |
| PUM1 | 0.34 |
| PVRIG | 0.368 |
| PXN | -0.3 |
| R3HDM2 | 0.325 |
| RAB1B | -0.302 |
| RAB31 | -0.309 |
| RAB3GAP1 | 0.304 |
| RAB40A | 0.346 |
| RAB9A | -0.357 |
| RABGAP1 | 0.323 |
| RABIF | -0.309 |
| RABL2A | 0.453 |
| RACGAP1 | 0.311 |
| RAD1 | 0.331 |
| RAD51 | 0.316 |
| RAD51AP1 | 0.353 |
| RAD52 | 0.381 |
| RAD54B | 0.386 |
| RAD54L2 | 0.325 |
| RAD9A | 0.313 |
| RAI1 | 0.362 |
| RALGAPB | 0.324 |
| RALGPS1 | 0.323 |
| RANBP10 | 0.376 |
| RANBP2 | 0.309 |
| RAPGEF2 | 0.379 |
| RASA1 | -0.349 |
| RBAK | 0.308 |
| RBBP6 | 0.313 |
| RBFOX2 | 0.3 |
| RBL2 | 0.306 |
| RBM12 | 0.318 |
| RBM12B | 0.412 |
| RBM14 | 0.372 |
| RBM14-RBM4 | 0.357 |
| RBM19 | 0.397 |
| RBM26 | 0.495 |
| RBM33 | 0.471 |
| RBM39 | 0.454 |
| RBM5 | 0.414 |
| RBM6 | 0.353 |
| RC3H1 | 0.404 |
| RCBTB1 | 0.374 |
| RCBTB2 | 0.453 |
| RCC2 | 0.303 |
| RCOR3 | 0.319 |
| RECQL5 | 0.372 |
| RERE | 0.355 |
| REV1 | 0.328 |
| RFC2 | 0.322 |
| RFWD3 | 0.313 |
| RFX1 | 0.302 |
| RGL2 | 0.315 |
| RGL3 | 0.367 |
| RGS12 | 0.312 |
| RGS17 | 0.353 |
| RGS4 | -0.312 |
| RHEB | 0.397 |
| RHO | 0.35 |
| RHOC | -0.336 |
| RIBC1 | 0.399 |
| RICTOR | 0.308 |
| RIN2 | -0.364 |
| RNF103-CHMP3 | 0.32 |
| RNF113B | 0.477 |
| RNF146 | 0.314 |
| RNF169 | 0.34 |
| RNF207 | 0.3 |
| RNF219 | 0.385 |
| RNF40 | 0.359 |
| RNF43 | 0.422 |
| RNF44 | -0.313 |
| RNMT | 0.304 |
| RNPC3 | 0.358 |
| ROMO1 | -0.304 |
| RPGR | 0.33 |
| RPL27A | -0.301 |
| RPL28 | -0.305 |
| RPL32 | -0.323 |
| RPL35 | -0.313 |
| RPL36 | -0.318 |
| RPL36A | -0.302 |
| RPL36AL | -0.364 |
| RPRD1B | 0.326 |
| RPRD2 | 0.314 |
| RPS14 | -0.319 |
| RPS27 | -0.304 |
| RREB1 | 0.339 |
| RRM2 | 0.3 |
| RSAD1 | -0.326 |
| RSPH4A | 0.385 |
| RTKN | 0.361 |
| RTP4 | -0.301 |
| RUFY3 | 0.367 |
| SAFB | 0.338 |
| SAP30BP | 0.308 |
| SAPCD1 | 0.378 |
| SAR1B | -0.307 |
| SATL1 | 0.304 |
| SBF1 | 0.308 |
| SBNO1 | 0.353 |
| SCAF8 | 0.328 |
| SCN11A | 0.356 |
| SCNN1D | 0.303 |
| SCYL3 | 0.318 |
| SDS | -0.332 |
| SEC16A | 0.337 |
| SEC24C | -0.374 |
| SEC31B | 0.427 |
| SEC61B | -0.354 |
| SEC61G | -0.334 |
| SECISBP2 | 0.354 |
| SECTM1 | -0.302 |
| SEMA3D | -0.303 |
| SEMA4C | 0.348 |
| SEMA4G | 0.312 |
| SENP7 | 0.37 |
| SERF2 | -0.31 |
| SERINC3 | -0.305 |
| SERP2 | 0.393 |
| SERPINF1 | -0.329 |
| SETD1A | 0.391 |
| SETD1B | 0.373 |
| SETD2 | 0.331 |
| SETD5 | 0.355 |
| SETDB1 | 0.363 |
| SETDB2 | 0.441 |
| SETX | 0.306 |
| SF1 | 0.38 |
| SF3B1 | 0.377 |
| SFPQ | 0.307 |
| SFSWAP | 0.367 |
| SGK2 | 0.429 |
| SGSM2 | 0.347 |
| SH2B1 | 0.368 |
| SH3BP5L | 0.318 |
| SHCBP1 | 0.361 |
| SHPRH | 0.341 |
| SIDT2 | -0.337 |
| SIN3B | 0.309 |
| SKA1 | 0.486 |
| SKI | 0.31 |
| SLC10A3 | -0.325 |
| SLC12A4 | 0.328 |
| SLC12A5 | 0.311 |
| SLC23A2 | 0.356 |
| SLC24A1 | 0.317 |
| SLC25A27 | 0.472 |
| SLC25A29 | 0.344 |
| SLC25A30 | 0.386 |
| SLC25A34 | 0.304 |
| SLC25A5 | -0.398 |
| SLC26A1 | 0.301 |
| SLC35E2B | 0.396 |
| SLC39A10 | 0.327 |
| SLC4A5 | 0.362 |
| SLC6A16 | 0.339 |
| SLC7A6 | 0.304 |
| SLC9A5 | 0.352 |
| SLC9A8 | 0.356 |
| SLCO6A1 | 0.333 |
| SLX4 | 0.369 |
| SLX4IP | 0.316 |
| SMARCC2 | 0.323 |
| SMC2 | 0.332 |
| SMC4 | 0.303 |
| SMCR8 | -0.369 |
| SMG1 | 0.396 |
| SMG6 | 0.349 |
| SMG7 | 0.355 |
| SMIM17 | 0.312 |
| SMPD4 | 0.325 |
| SMS | 0.316 |
| SNAI1 | -0.309 |
| SNAI2 | -0.359 |
| SNAPC4 | 0.334 |
| SNRNP200 | 0.358 |
| SNRNP70 | 0.375 |
| SNRPF | 0.378 |
| SNX12 | -0.331 |
| SOCS7 | 0.353 |
| SOGA1 | 0.38 |
| SOWAHB | -0.303 |
| SOX11 | 0.321 |
| SOX17 | 0.37 |
| SOX6 | 0.34 |
| SPARC | -0.319 |
| SPATA12 | 0.392 |
| SPC24 | 0.474 |
| SPC25 | 0.458 |
| SPDYA | 0.413 |
| SPDYE1 | 0.422 |
| SPEF2 | 0.303 |
| SPEN | 0.391 |
| SPERT | 0.305 |
| SPG7 | 0.336 |
| SPINK5 | 0.317 |
| SPP1 | -0.315 |
| SPR | -0.312 |
| SPTBN1 | 0.309 |
| SPTBN2 | 0.327 |
| SPTSSA | 0.313 |
| SRCAP | 0.404 |
| SRD5A3 | -0.337 |
| SREK1 | 0.357 |
| SRGAP3 | 0.336 |
| SRP68 | 0.303 |
| SRPRB | -0.37 |
| SRRM1 | 0.302 |
| SRRM2 | 0.403 |
| SRSF2 | 0.375 |
| SRSF5 | 0.41 |
| SRSF6 | 0.322 |
| SS18L1 | 0.349 |
| ST6GALNAC2 | 0.324 |
| STAG1 | 0.304 |
| STARD9 | 0.353 |
| STAT5B | -0.314 |
| STK35 | 0.369 |
| STK36 | 0.456 |
| STMN1 | 0.365 |
| STOX2 | 0.338 |
| STRADA | 0.426 |
| STX16 | 0.316 |
| STX1A | 0.306 |
| STXBP4 | 0.313 |
| SUB1 | 0.36 |
| SUCLA2 | 0.344 |
| SUGP2 | 0.4 |
| SUGT1 | 0.484 |
| SULT1C4 | 0.34 |
| SUMO1 | 0.304 |
| SUN1 | 0.305 |
| SUPT20H | 0.413 |
| SUPT6H | 0.303 |
| SVEP1 | -0.307 |
| SYCP2L | 0.304 |
| SYK | 0.336 |
| SYNGAP1 | 0.424 |
| SYNGR2 | -0.307 |
| SYNRG | 0.309 |
| SZT2 | 0.313 |
| TAC4 | 0.322 |
| TACC3 | 0.347 |
| TAF1 | 0.35 |
| TAF10 | -0.3 |
| TAF13 | 0.31 |
| TAF1C | 0.333 |
| TAF4 | 0.327 |
| TAGLN2 | -0.381 |
| TANC1 | 0.347 |
| TANC2 | 0.339 |
| TAOK1 | 0.346 |
| TAOK2 | 0.367 |
| TAP1 | -0.321 |
| TARBP1 | 0.311 |
| TARDBP | 0.328 |
| TAS2R20 | 0.406 |
| TAS2R5 | 0.47 |
| TBC1D14 | -0.388 |
| TBC1D20 | 0.386 |
| TBC1D2B | -0.309 |
| TBC1D9B | -0.337 |
| TCERG1 | 0.309 |
| TCF20 | 0.312 |
| TCP11L2 | 0.322 |
| TCTN3 | -0.315 |
| TDRD3 | 0.406 |
| TEAD1 | 0.308 |
| TEC | 0.308 |
| TEDDM1 | 0.305 |
| TET2 | 0.382 |
| TET3 | 0.416 |
| TFAP2C | -0.302 |
| TGFBI | -0.343 |
| TGFBR2 | -0.314 |
| TGFBRAP1 | 0.339 |
| THOC7 | -0.31 |
| THRAP3 | 0.323 |
| TIA1 | 0.384 |
| TIMM23 | 0.397 |
| TIMP3 | -0.332 |
| TJAP1 | 0.31 |
| TK1 | 0.385 |
| TLK2 | 0.315 |
| TLR9 | 0.312 |
| TM9SF4 | 0.304 |
| TMA7 | 0.368 |
| TMCC1 | 0.323 |
| TMEM120B | 0.325 |
| TMEM132B | 0.32 |
| TMEM143 | 0.309 |
| TMEM200A | -0.324 |
| TMEM231 | 0.306 |
| TMEM59 | -0.304 |
| TMEM70 | -0.325 |
| TMEM80 | 0.337 |
| TMEM91 | 0.426 |
| TMPRSS11F | 0.332 |
| TMPRSS3 | 0.397 |
| TMSB4X | -0.305 |
| TMTC4 | 0.316 |
| TMX2 | -0.313 |
| TNFRSF1B | -0.3 |
| TNFRSF25 | 0.314 |
| TNK2 | 0.375 |
| TNKS | 0.3 |
| TNKS2 | 0.323 |
| TNRC18 | 0.378 |
| TNRC6A | 0.439 |
| TNRC6B | 0.414 |
| TOB2 | 0.325 |
| TOP2A | 0.386 |
| TOR3A | -0.314 |
| TP73 | 0.385 |
| TPCN2 | 0.393 |
| TPH1 | 0.337 |
| TPM3 | -0.326 |
| TPP2 | 0.326 |
| TPR | 0.308 |
| TPX2 | 0.419 |
| TRAM2 | 0.316 |
| TRIB2 | -0.3 |
| TRIM13 | 0.505 |
| TRIM25 | 0.342 |
| TRIM45 | 0.319 |
| TRIM46 | 0.345 |
| TRIM52 | 0.326 |
| TRIM66 | 0.356 |
| TRIM73 | 0.32 |
| TRIM74 | 0.321 |
| TRIO | 0.334 |
| TRIP13 | 0.376 |
| TRMT10B | 0.334 |
| TRO | 0.308 |
| TRPV1 | 0.356 |
| TRRAP | 0.354 |
| TSC1 | 0.378 |
| TSC22D1 | 0.346 |
| TSGA10 | 0.337 |
| TSPAN32 | 0.319 |
| TSPYL2 | 0.309 |
| TSPYL4 | 0.345 |
| TSTD2 | 0.371 |
| TTC14 | 0.42 |
| TTC28 | 0.384 |
| TTC29 | 0.329 |
| TTLL3 | 0.454 |
| TTLL9 | 0.378 |
| TTPAL | 0.307 |
| TUB | 0.315 |
| TUBE1 | 0.326 |
| TVP23C | 0.314 |
| TXNDC17 | -0.32 |
| TXNDC5 | -0.367 |
| U2AF1L4 | 0.306 |
| UBAP1L | 0.374 |
| UBE2C | 0.445 |
| UBE2L6 | -0.389 |
| UBE2S | 0.409 |
| UBE2V1 | 0.324 |
| UBE3B | 0.33 |
| UBE4B | 0.352 |
| UBFD1 | 0.327 |
| UBL5 | -0.344 |
| UBN1 | 0.342 |
| UBN2 | 0.358 |
| UBR1 | 0.314 |
| UBR4 | 0.372 |
| UBTF | 0.349 |
| UBXN10 | 0.355 |
| UBXN2B | 0.339 |
| UBXN7 | 0.347 |
| UCHL3 | 0.337 |
| ULK1 | 0.408 |
| UNC119B | 0.308 |
| UNK | 0.317 |
| UNKL | 0.406 |
| UPF3A | 0.477 |
| UQCC1 | 0.312 |
| UQCR11 | -0.348 |
| UQCRQ | -0.37 |
| USP10 | 0.391 |
| USP22 | 0.314 |
| USP32 | 0.302 |
| USP34 | 0.386 |
| USP36 | 0.344 |
| USP37 | 0.338 |
| USP42 | 0.311 |
| USP49 | 0.327 |
| USPL1 | 0.328 |
| UTP14C | 0.425 |
| UVSSA | 0.394 |
| VAMP8 | -0.329 |
| VANGL2 | 0.323 |
| VCAM1 | -0.32 |
| VCAN | -0.33 |
| VGLL4 | -0.375 |
| VPS11 | -0.325 |
| VPS13B | 0.323 |
| VPS29 | 0.3 |
| VPS36 | 0.48 |
| VPS39 | 0.337 |
| VRK1 | 0.329 |
| VWA3A | 0.393 |
| VWA8 | 0.355 |
| WAC | 0.322 |
| WARS | -0.313 |
| WBP4 | 0.488 |
| WDFY2 | 0.468 |
| WDFY3 | 0.325 |
| WDR19 | 0.356 |
| WDR26 | 0.301 |
| WDR27 | 0.498 |
| WDR35 | 0.385 |
| WDR59 | 0.324 |
| WDR6 | 0.333 |
| WDR63 | 0.337 |
| WDR73 | 0.352 |
| WDR81 | -0.318 |
| WEE2 | 0.389 |
| WRN | 0.305 |
| WSB1 | 0.318 |
| WTIP | 0.363 |
| XPC | 0.302 |
| XPO4 | 0.312 |
| XRCC2 | 0.361 |
| YEATS2 | 0.371 |
| YIPF1 | -0.311 |
| YLPM1 | 0.345 |
| YPEL4 | 0.349 |
| YTHDC1 | 0.312 |
| ZACN | 0.315 |
| ZBTB12 | 0.317 |
| ZBTB3 | 0.305 |
| ZBTB34 | 0.316 |
| ZBTB37 | 0.409 |
| ZBTB39 | 0.339 |
| ZBTB40 | 0.423 |
| ZBTB46 | 0.345 |
| ZBTB8OS | 0.32 |
| ZC2HC1C | 0.344 |
| ZC3H11A | 0.383 |
| ZC3H12B | 0.357 |
| ZC3H13 | 0.549 |
| ZC3H6 | 0.307 |
| ZC3H7B | 0.387 |
| ZCCHC14 | 0.303 |
| ZCCHC18 | 0.315 |
| ZCCHC24 | -0.322 |
| ZCCHC8 | 0.303 |
| ZDHHC17 | 0.332 |
| ZDHHC8 | 0.304 |
| ZEB1 | -0.323 |
| ZFC3H1 | 0.36 |
| ZFHX3 | 0.32 |
| ZFP2 | 0.31 |
| ZFP64 | 0.309 |
| ZFYVE26 | 0.324 |
| ZKSCAN1 | 0.335 |
| ZKSCAN2 | 0.311 |
| ZKSCAN7 | 0.364 |
| ZKSCAN8 | 0.364 |
| ZMIZ1 | 0.313 |
| ZMPSTE24 | -0.352 |
| ZMYM2 | 0.355 |
| ZMYND10 | 0.372 |
| ZNF10 | 0.388 |
| ZNF106 | 0.304 |
| ZNF117 | 0.302 |
| ZNF142 | 0.338 |
| ZNF154 | 0.305 |
| ZNF160 | 0.334 |
| ZNF169 | 0.364 |
| ZNF2 | 0.343 |
| ZNF23 | 0.425 |
| ZNF236 | 0.421 |
| ZNF248 | 0.354 |
| ZNF25 | 0.309 |
| ZNF254 | 0.321 |
| ZNF26 | 0.368 |
| ZNF263 | 0.315 |
| ZNF264 | 0.313 |
| ZNF286A | 0.318 |
| ZNF292 | 0.321 |
| ZNF318 | 0.349 |
| ZNF319 | 0.309 |
| ZNF333 | 0.363 |
| ZNF334 | 0.338 |
| ZNF335 | 0.377 |
| ZNF337 | 0.492 |
| ZNF33A | 0.375 |
| ZNF367 | 0.341 |
| ZNF37A | 0.313 |
| ZNF384 | -0.302 |
| ZNF407 | 0.362 |
| ZNF417 | 0.321 |
| ZNF423 | 0.306 |
| ZNF440 | 0.303 |
| ZNF445 | 0.375 |
| ZNF471 | 0.315 |
| ZNF483 | 0.348 |
| ZNF493 | 0.316 |
| ZNF496 | 0.359 |
| ZNF497 | 0.305 |
| ZNF500 | 0.305 |
| ZNF512 | 0.349 |
| ZNF512B | 0.387 |
| ZNF514 | 0.44 |
| ZNF516 | 0.311 |
| ZNF517 | 0.335 |
| ZNF519 | 0.351 |
| ZNF528 | 0.322 |
| ZNF532 | 0.355 |
| ZNF546 | 0.341 |
| ZNF550 | 0.331 |
| ZNF554 | 0.345 |
| ZNF558 | 0.331 |
| ZNF577 | 0.311 |
| ZNF587 | 0.36 |
| ZNF589 | 0.315 |
| ZNF592 | 0.314 |
| ZNF594 | 0.316 |
| ZNF605 | 0.396 |
| ZNF606 | 0.351 |
| ZNF609 | 0.322 |
| ZNF618 | 0.391 |
| ZNF621 | 0.322 |
| ZNF629 | 0.41 |
| ZNF638 | 0.338 |
| ZNF646 | 0.388 |
| ZNF660 | 0.395 |
| ZNF662 | 0.349 |
| ZNF667 | 0.302 |
| ZNF668 | -0.31 |
| ZNF687 | 0.312 |
| ZNF692 | 0.436 |
| ZNF699 | -0.322 |
| ZNF70 | 0.374 |
| ZNF711 | 0.314 |
| ZNF713 | 0.352 |
| ZNF736 | 0.321 |
| ZNF764 | 0.348 |
| ZNF770 | 0.326 |
| ZNF783 | 0.35 |
| ZNF785 | 0.373 |
| ZNF8 | 0.39 |
| ZNF81 | 0.355 |
| ZNF827 | 0.409 |
| ZNF83 | 0.319 |
| ZNF84 | 0.371 |
| ZNF841 | 0.354 |
| ZNF853 | 0.302 |
| ZNHIT1 | -0.319 |
| ZRANB2 | 0.308 |
| ZSCAN20 | 0.32 |
| ZSCAN23 | 0.351 |
| ZSCAN25 | 0.311 |
| ZSCAN29 | 0.352 |
| ZSCAN30 | 0.311 |
| ZSWIM1 | 0.305 |
| ZSWIM8 | 0.331 |
| ZWILCH | 0.312 |
| ZWINT | 0.402 |
| ZXDC | 0.421 |
| ZZEF1 | 0.307 |
