# Supplementary figures and images for: Identification and validation of a five-lncRNA signature for predicting survival with targeted drug candidates in ovarian cancer
Source: Bioengineered. 2021 Jul 5;12(1):3263–74. doi: 10.1080/21655979.2021.1946632 (PMC8806566; doi:10.1080/21655979.2021.1946632)

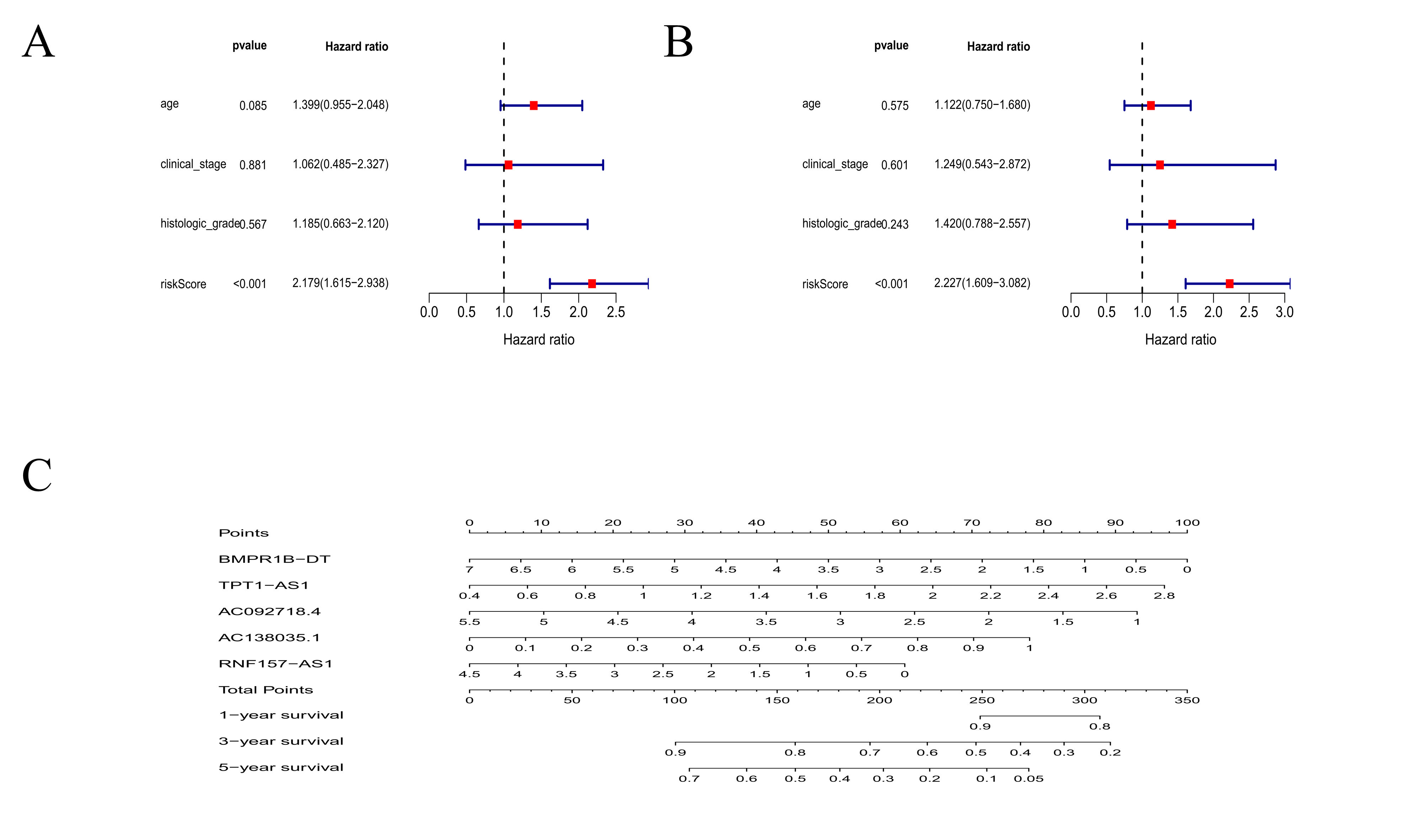

Supplement: Supplemental Material [file KBIE_A_1946632_SM7043.zip › supplementary/FIG S1.tif]

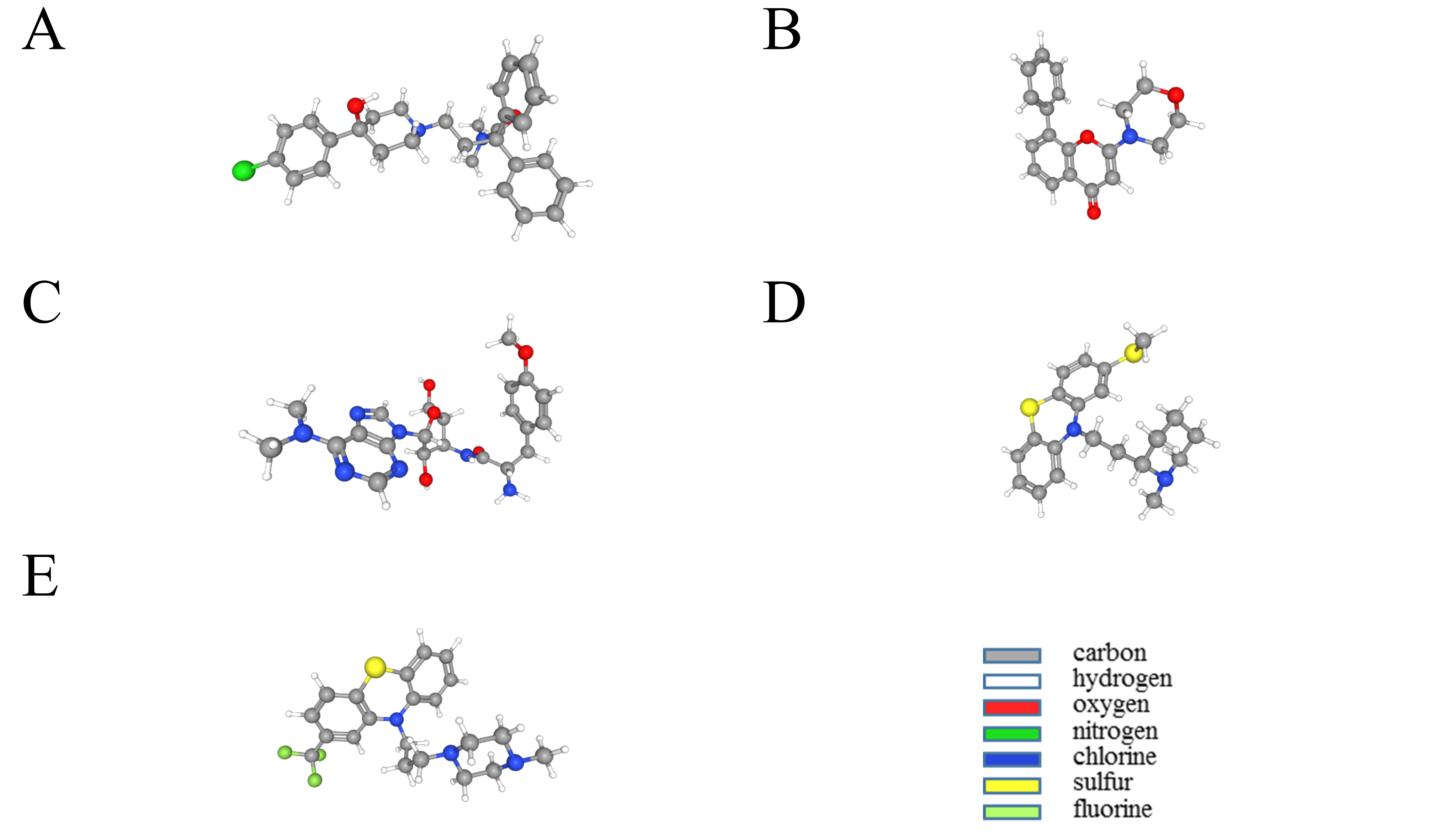

Supplement: Supplemental Material [file KBIE_A_1946632_SM7043.zip › supplementary/FIG S2.tif]
